# Micro/nanoplastics: from synthesis to assimilation, global environmental impacts, challenges, consequences, and advanced remediation strategies

**DOI:** 10.1039/d6ra08464h

**Published:** 2026-10-19

**Authors:** Muhammad Rizwan, Amir Zada, Ghulam Murtaza, Nolubabalo Matinise, Hong Chen

**Affiliations:** a Key Laboratory of Water-Sediment Sciences and Water Disaster Prevention of Hunan Province, School of Hydraulic and Environmental Engineering, Changsha University of Science & Technology Changsha 410114 China chenh@csust.edu.cn; b Department of Environmental Engineering, Faculty of Civil Engineering, Yildiz Technical University Esenler Istanbul 34220 Türkiye; c Department of Chemistry, Abdul Wali Khan University Mardan Khyber Pakhtunkhwa 23200 Pakistan; d UNESCO-UNISA Africa Chair in Nanosciences and Nanotechnology, College of Graduate Studies, University of South Africa Muckleneuk Ridge, PO Box 392 Pretoria 0002 South Africa matinn@unisa.ac.za; e School of Agriculture, Yunnan University Kunming 650504 Yunnan China

## Abstract

Environmental pollution has become a hot research topic and a grave issue throughout the world. The presence of plastic and its derivatives almost everywhere and in every segment of the environment has polluted water (the marine environment), soil, and even the atmosphere. Plants, animals and microorganisms are all equally affected. In this review article, we have discussed in detail the applications of plastics, especially micro/nanoplastics, their global distribution and their uses. The aging of plastic and its consequences on the physical and chemical properties of plastic-based materials is discussed. The effects of micro/nanoplastics on water, soil, plants, crop productivity, and animals (both aquatic and terrestrial) have been discussed in detail. Recycling of plastics (physical and chemical) and techniques for their eradication, including physical, biological and advanced chemical methods, to cope with plastic-based pollution have been discussed. The theory and mechanisms behind the biotic removal, adsorption, density separation, ultrafiltration, froth floatation, ozonation, coagulation, electrocatalytic oxidation and photocatalytic oxidation of micro/nanoplastics to deal with plastic pollution and its impacts properly have been discussed in detail. Although several review articles on the eradication of plastics have been published, and a huge amount of research work to reduce the impacts of plastic-based pollution is underway, the eradication mechanisms are still in the developmental stages and systematic debates are lacking. This review article presents a detailed discussion of the recent expansions in the removal of plastic-based materials and their upcycling into energy-rich compounds. The role of biochar in the removal of plastic-based materials is also debated. Finally, future perspectives and research gaps in the field of plastic-based pollution and the removal of micro/nanoplastics from various sectors of the environment are presented.

## Abstract 2

Plastic waste is collected, sorted, and then processed into plastic monomers through chemical recycling. These monomers are used to produce new polymers and cellular materials.

## Graphical abstract

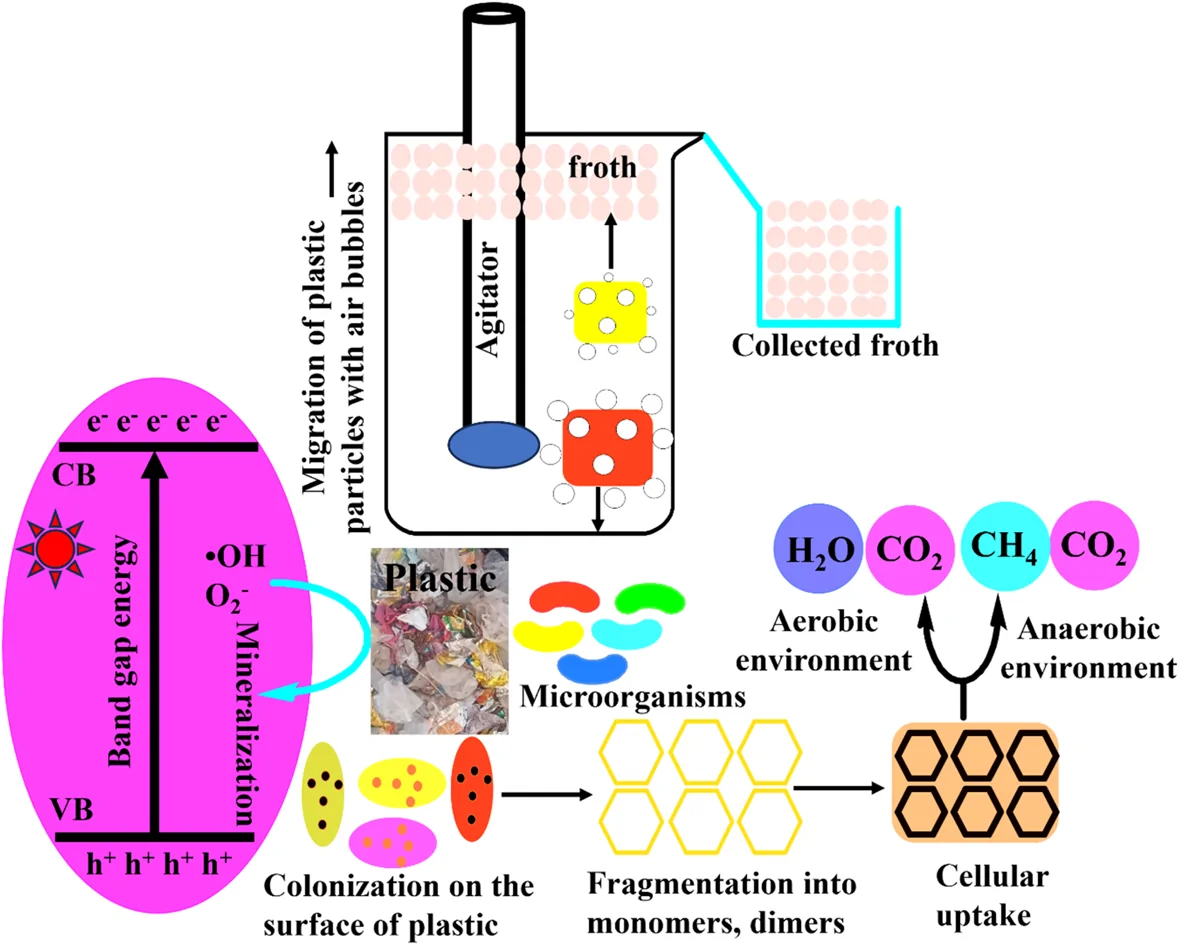

## 1 Introduction

The deterioration of the environment due to the presence of various hazardous pollutants is a major issue currently. Many toxic organic and inorganic pollutants with diverse chemical natures have caused major damage to the environment.^1^ The combination of solid, liquid and gaseous effluents from manufacturing industries, hospitals, municipal committees, and agricultural sites has intensified and amplified the destruction of the environment. Synthetic dyes, heavy metals, pharmaceutical products, electronic wastes, crop production activators, poultry farm wastes, nuclear materials and numerous household wastes with significant amounts of toxic materials have impacted the environment greatly.^2^ The accumulation of complex-structured antibiotics has intensified the growth of drug-resistant microbes, with scores of deaths even in developed countries such as the USA, China, Germany, France, *etc.* Recently, many agricultural products such as wheat and rice in China have been reported to contain excessive amounts of As, a carcinogenic element whose amount increases with increasing global temperature. Every year, huge quantities of electronic wastes, such as mobile and laptop batteries, are discharged without proper recycling and disposal, causing severe environmental issues.

Plastic has become a crucial part of our lives, leading to an extraordinary surge in its production and use in the last few decades. However, its mismanagement has led to widespread environmental concerns. Recently, the accumulation of micro/nanoplastics in water, soil, and even the bodies of animals, plants and microorganisms has become a new source of toxic materials with significant concerns.^3^ Plastic-based pollution is found on every continent, from land to deep oceans and from water to land and mountains. Since 1940, the global production of plastics and their products has climbed to nearly 368 million metric tons in 2019 and 400.30 million tons in 2022, indicating a rapid increase in their utilization and production. More discouragingly, only 9% of the produced plastic was recycled; 12% was incinerated and the remaining 79% was dumped into the natural environment as a landfill, resulting in unmanageable practices to cope with plastic-based pollution. Plastic pollution may create large visible particles, as well as micro/nanoplastics, which can penetrate the cells and tissues of animals and plants, resulting in various diseases. Plastic-based materials are ideal surfaces for the adsorption and retention of numerous hazardous and toxic organic and inorganic pollutants and heavy metals. The animals frequently affected are molluscs, seabirds, fishes, turtles, marine mammals, and crustaceans, with observed effects of increasing physiological damage, largely related to metabolic and ingestion activity. In addition, micro/nanoplastics cause genetic and immunological alternations, gastrointestinal issues, reduced growth, low fertility, and premature mortality. Study shows that both acute and chronic responses to micro/nanoplastics exposure result in disruptions to the reproductive system, immunity, and the endocrine system. The uptake of micro/nanoplastics by microorganisms highly facilitates the dispersal of potentially invasive species, which threaten additional problems in the ecosystem.^4–6^

The above-mentioned hazards of microplastics demand two immediate actions: (i) the restriction of the synthesis of plastic and its products by replacing them with cost-effective naturally produced materials, and (ii) the employment of proper techniques to remove the microplastics already distributed in different sectors of the environment. Various attempts have been made to achieve the removal of microplastic-based toxic pollutants such as adsorption, burning, filtration, electrocoagulation, and flocculation, biodegradation, and advanced oxidation processes. Although all these processes are effective in the removal of microplastics from soil and water, many of the mentioned methods are costly and have low removal efficiencies. Photocatalysis and advanced oxidation techniques are exceptionally effective in dealing with pollutants of diverse natures. In particular, the application of biochar in the removal of microplastics is exceptionally cost-effective, with excellent eradication efficiencies.^7^

Plastic-based pollution is a critical issue and requires sustainable solutions. Many review articles have reported on eradication techniques for plastic-based materials; however, systematic debates on the mechanisms of micro/nanoplastic removal are lacking. Although plastics demonstrate significant health and environmental issues, a significant gap still exists in the published literature. While a huge amount of research work is underway across the world to reduce the impacts of plastic-based pollution, the eradication mechanisms are still in the developmental stages. This article integrates state-of-the-art multidisciplinary techniques for the eradication of plastic-based pollutants and gives research directions for upcycling, highlighting the need for advanced engineering materials to deal with plastic-based pollution. It presents a detailed discussion on the recent expansions in the removal of plastic-based materials and their upcycling into energy-rich compounds. It provides a useful source of knowledge for researchers employed in the field of plastic-based pollution and delivers insights into the existing state of research on micro/nanoplastics, their distribution on land and in water, associated hazards, and effective remediation techniques. Each section is supported with suitable figures. We hope that this article will aid in the comprehension of microplastic-based pollution and its effective removal.

## 2 Uses of plastics

Over the last 50 years, the production of plastic has increased exponentially to several million tons annually. The name plastic is derived from the Greek word “plastikos”, which reflects its capability to be molded and shaped into lightweight, durable and cost-effective materials easily. It is a high-molecular-mass polymer synthetically obtained from the polymerization of small units (monomers) of natural origin (petroleum).^8^ Although Bakelite, a type of plastic, has been known for more than a hundred years, the bulk production of plastic and its derivatives launched in the mid-20th century. As a result, a plethora of versatile compositions resulted in the formation of lightweight and durable materials with diverse applications for modernization and progress in varied fields, including the automobile industry, electronic devices, cables, food preservation, packaging materials, construction, energy generation, healthcare, domestic products and home appliances. Due to its low density, excellent plasticity, poor electric and heat conductivity, remarkable resistance, and stability, it is a common material for the fabrication of numerous household (bags, carpets, containers, decoration pieces, *etc.*) and industrial products.^9,10^ Plastic and its products have restructured many industrial products. They have almost completely revolutionized and modernized life. However, this revolution and modernization have fostered a “use and throw away” attitude in ordinary people, resulting in severe pollution with the accumulation of huge quantities of plastics in the soil and water.^11^ Their large-scale use in disposable form, including packaging substances, results in their build-up in the environment, as indicated by the nearly 10% mass content of plastic in global municipal garbage. Although various attempts have been made to overcome their accumulation in the environment, these attempts are inadequate.^12^Fig. 1 shows the presence of plastics in various sectors of the environment and their applications.^13^

**Fig. 1:**
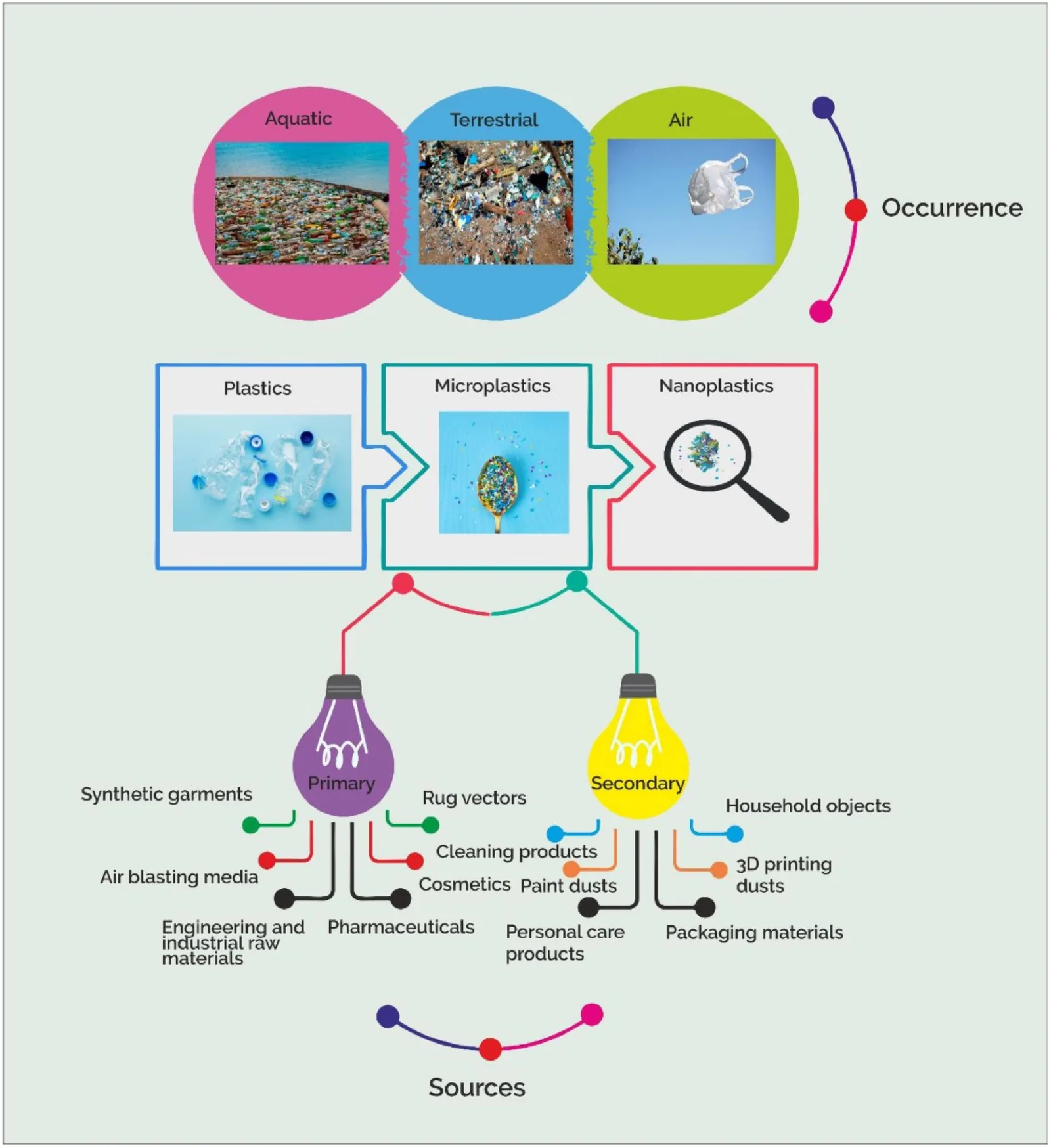
An overview of the occurrence and sources of microplastics in the ecosystem. Figure adapted from ref. 13 with permission from Elsevier, copyright 2025 under License Number: 6340641015657.

## 3 What are micro/nanoplastics?

Various terms, such as nano-, micro-, and macro-, are used to describe plastics. This terminology emerged recently after research on plastic-based pollution. There is strong debate regarding the upper limit of nanoplastics (<1 µm or <100 nm), and a need for terms such as mesoplastics (5–25 mm). However, it is generally accepted amongst the research community that microplastics are plastic particles with a size of less than 5 mm, while nanoplastics are particles with a size of less than 1 µm morphologically characterized as fragments, films, fibres and foams.^14,15^ The intensity of the pollution caused by various types of plastics, such as low/high-density polyethylene, polystyrene, polypropylene, polyethylene terephthalate and polyvinyl chloride, is measured in terms of the degree of contamination from their micro/nanoplastics found in soil and water.^16^ Microplastics are further classified as primary and secondary microplastics. Primary microplastics are deliberately produced particles for use in personal care items including antiaging agents, toothpastes, sunscreens and facial scrubbers, skin creams and body washes, hair dyes, and nail polishes.^17^ They are also generated from debris such as particulate emission from industrial synthesis, and particles produced during the wear and tear (*i.e.*, of rubber tires on the road) and conservation of plastic materials. Secondary microplastics result from the weathering and physical abrasion of bulk plastics. Owing to their tiny dimensions, these particles are exceptionally toxic. It is estimated that microplastics are more hazardous than bulk plastics, and significantly endanger the existence of terrestrial and ocean life. The sources generating and adding microplastics to the environment include effluents of particulates from plastic manufacturing industries, and particles released to the atmosphere during the wear and tear of plastic products. Huge quantities of microplastics are added to the soil and bodies of water during the plastic manufacturing stage through the washing, cutting, painting, molding and polishing of various products.^18^Fig. 2 shows the annual global production of plastic and its effects.^19^

**Fig. 2:**
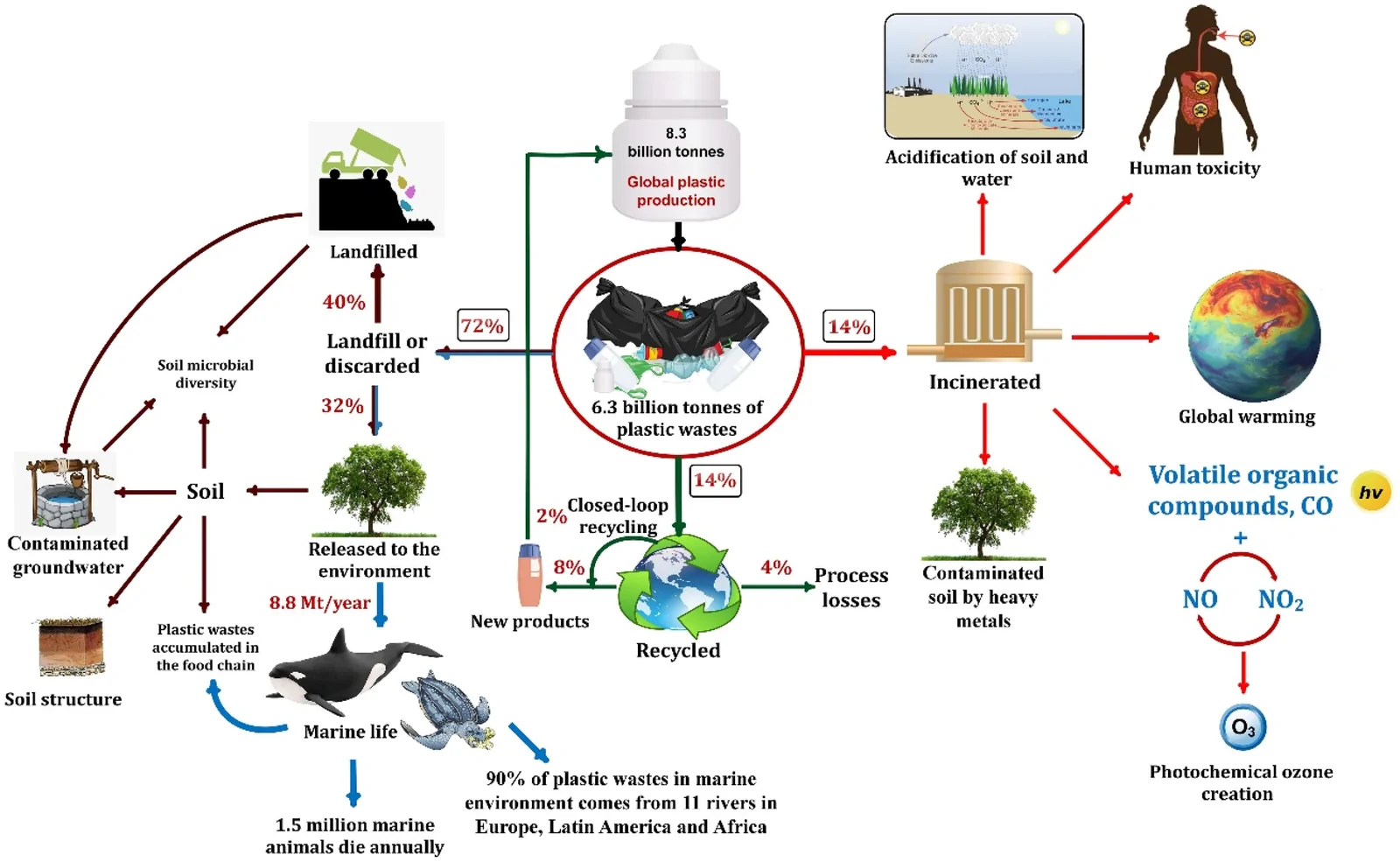
Schematic showing the fate of plastics and their hazardous effects on environment. Figure adapted from ref. 19 with permission from Elsevier, copyright 2025 under License Number: 6340641325457.

## 4 Microplastics in the environment

### 4.1 Microplastics in the marine environment

Analogous to the ice age experienced by the Earth, we are now in the plastic age. Plastic and its derivatives, such as microplastics and nanoplastics, are found everywhere in the environment.^20^ Almost every place on the globe has been found to contain plastic and its constituents. Plastics have been found in nearly hundred lakes worldwide, with loads ranging from 0.27 to 34 000 counts per m^3^ and 10.9 × 10^2^–2.09 × 10^4^ counts per km^2^. Therefore, our planet is approaching environmental devastation and catastrophe. Numerous lakes are shrinking, lake salinization is increasing, and water quality has deteriorated over the last 30 years due to the combination of climatic effects and human activities.^21^ Every year, huge amounts of plastic and its various products are synthesized around the world for various purposes. Their unstoppable and continuous fabrication has resulted in the addition of huge quantities of microplastics into various bodies of water, which are becoming a dumping ground for plastic garbage. Bulk plastic and microplastics are now universal in the marine environment, where they are seen everywhere from shorelines to the deep-sea environment. The accumulation of plastic garbage in the oceans is therefore a budding environmental issue. Owing to our immense dependence on plastic products, most of these materials are designed as disposable products for single use. Subsequently, they are dumped and discarded into the external environment.^22^ Data analysis confirms that a huge increase in the total use of plastic was observed between 1990 and 2000, and the amount of plastic produced in 2000 was almost double that generated in 1990. Although a 3.7-fold increase in the production of plastic occurred up to 2019, the projected forecast demonstrates a more reasonable average increase of 1.7-fold to 2050.^23^ From extraction to preparation, the whole lifecycle of microplastics creates ecological concerns. The accumulation of microplastics and nanoplastics in the marine environment is shocking, with an estimated 5 trillion particles floating in bodies of water and about 12.7 million metric tons entering the oceans annually.^9^

### 4.2 Microplastics in soil and land

Globalization, floods, and air currents are the major agents that redistribute and restructure microplastics on land and water, leading to their presence in environments ranging from land to oceans and from land to mountains.^24^ Various human activities are responsible for the accumulation of plastic and its derivatives in soil, but principally, the improper dumping of plastic-based garbage and careless use of plastic-based materials in agriculture, the automobile industry, and construction are the chief sources, contributing huge quantities of plastics to soil pollution. The primary sources responsible for the distribution of micro/nanoplastics in soil include wastewater irrigation and fragmentation of plastic-based materials in landfills.^25,26^ The application of fertilizers and disintegration of the tyres of cars also make significant contributions. Ashes from incineration sites, packing materials from shops and hotels, and plastic bottles from cold drinks and other industries are amongst the sources of plastic and its derivatives.^27^ Finishing processes in the textile and fabric industries contribute huge quantities of microplastics to the environment. Similarly, automobiles, construction sites, consumer goods, electrical and electronic devices (so-called e-wastes), medical wastes, and food packaging sites also contribute a measurable quantity of microplastics to the soil.^28^ The life cycle of plastic, with its processing, distribution and disposal into the environment, is shown in Fig. 3.^29^

**Fig. 3:**
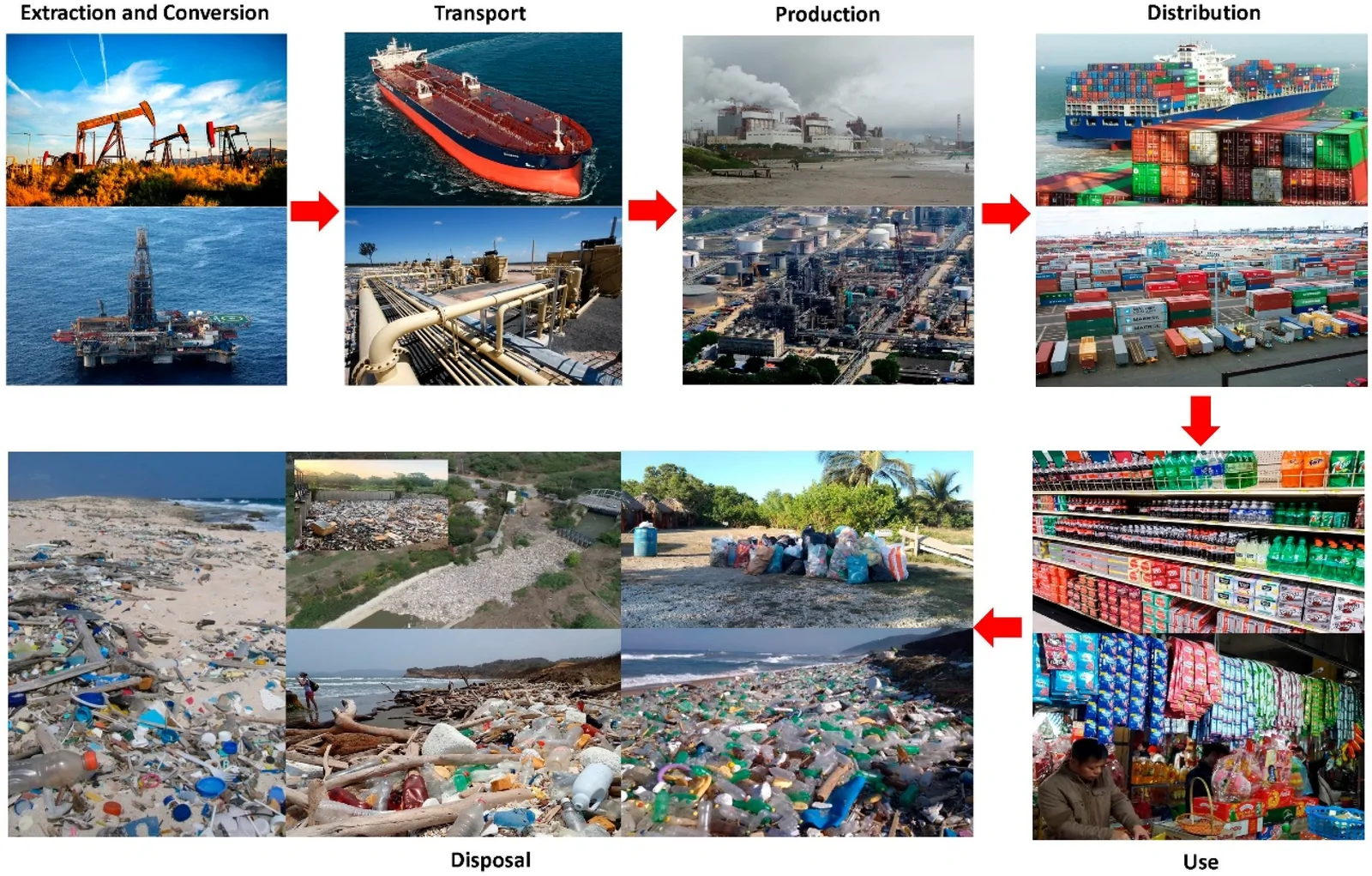
Plastic, from birth to disposal and escape into the environment. Figure adapted from ref. 29 with permission from Elsevier, copyright 2025 under License Number: 6340650029099.

## 5 What is the aging of plastics?

Plastic is deliberately shaped into different designs for various applications. After their birth (preparation/synthesis), the aging process of plastics and microplastics starts upon exposure to the environment. Their lateral and vertical distribution over considerable distances through environmental agents such as water and wind, along with numerous human activities, and their exposure to mechanical disintegration, photothermal radiation, and biodegradation determine their physical age.^30,31^ The disintegration and fragmentation of aged plastic is becoming a cause of huge concern, as it determines the magnitude of toxicity associated with plastic pollution globally. The changes that take place in the integrity of plastic are proportional to its age. The most important is the physical aging of plastic; during which rapid fragmentation takes place, which increases the amount of micro/nanoplastics in the environment. The surface morphology of plastic changes significantly with its aging, and the size of the micro/nanoplastic particles decreases drastically. A rougher morphology is established, with the generation of various cracks, flaws, fractures, defects, and pores and the formation of numerous small debris and fragments as exposure to mechanical abrasion and ultraviolet radiation is prolonged. With aging, the volume, pore structure, and surface chemistry of micro/nanoplastics are significantly modulated; subsequently, their transport and distribution in the environment are affected.^32,33^ Enhancement in surface area with aging results in the adhesion of complex communities of microbial colonies, including autotrophic, heterotrophic, and symbiotic microbes, which are generally referred to as the plastisphere, to the surface of plastic, improving its hydrophilicity and density through biofilm formation. The changes in hydrophilicity and wettability due to biofilm formation on the surface of plastics accelerate their sinking into the beds of oceans, making their removal more difficult. With exposure to intense ultraviolet radiation and chemical reactions occurring under high temperature and microbially colonized conditions, changes in elemental composition are prevalent. The oxygen content is strongly affected, and significant changes take place in the polymeric chains as physical aging increases.^34^ The fate of microplastics in the environment is related to their structures and morphologies. Plastics with more amorphous character are degraded into microplastics faster than semi-crystalline plastics with more ordered structures. The loss in the molecular mass (due to changes in elemental composition) of certain amorphous plastics such as polyvinyl chloride and polycarbonate after aging is related to a reduction in transition temperature, as these plastics resist high-temperature conditions owing to their exceptionally high transition temperature. As a result, fragmentation of the long polymeric chains takes place, resulting in the formation of micro/nanoplastics in the environment. The physical properties of aged plastic suffer dramatic changes owing to the changes in the carbon chains. This highly facilitates the fragmentation of plastics and the leakage and discharge of additives to create economic and toxicological threats to animals and the environment. Aged micro/nanoplastics interact readily with organic matter (humic acid, cellulose, *etc.*) and extracellular materials secreted by microbes to the environment. This causes significant changes in the bioreactivity, chemical stability and physical structures of the microplastics. The adsorption of hazardous pollutants and heavy metals is improved with prolonged aging.^35^

### 5.1 Aging mechanism

The mechanism of aging depends on the nature of the plastic and the nature of the degrading species. Micro/nanoplastics undergo aggressive aging stimulated by various advanced oxidation processes such as Fenton-, persulfate- and ozone-based oxidation. Advanced oxidation processes such as UV/chlorine, UV/H_2_O_2_, UV/persulfate, and O_3_/H_2_O_2_ processes cause rapid aging, while Fenton treatment causes a considerable degree of aging. Advanced-oxidation-mediated aging is stimulated by UV light and reactive oxygen species generation. Exposure to UV light induces surface cracking in plastic and speeds up the generation of reactive oxygen species to promote aging. The process of aging comprises initiation, propagation, and termination steps. The aging process is initiated by UV light, which excites chromophores and generates an excited singlet state. The excited singlet state undergoes intersystem crossing and converts into a triplet state. As a result, energy is transferred to adjacent bonds (C–H/C–C), resulting in the production of alkyl free radicals (R˙). The formed R˙ reacts with O_2_ to yield peroxy radicals (ROO˙), leading to the onset of oxidation.^36,37^ The propagation step further augments oxidation through autoxidation. Peroxy radicals remove hydrogen from the polymeric backbone to form hydroperoxides. The decomposition of hydroperoxides under UV light yields highly reactive alkoxy free radicals (RO˙) and ˙OH. Further abstraction of hydrogen from the polymeric structure through RO˙ and ˙OH may result in the formation of alcohol or aldehydes and ketones, as shown in Fig. 4a. These secondary compounds are further oxidized into carboxylic acids and esters. In the final termination step, the generated reactive free radicals combine with each other or stabilizing agents in plastic to generate nonradical stable products and complete the aging process. UV light also activates H_2_O_2_, chlorine, and persulfates, producing the reactive species ˙OH, O_2_˙^−^, SO_4_˙^−^, Cl˙ and ClO˙. Due to these reactive species, plastic undergoes rapid aging and decomposition *via* chain scission into low-molecular-mass compounds.^38,39^

**Fig. 4:**
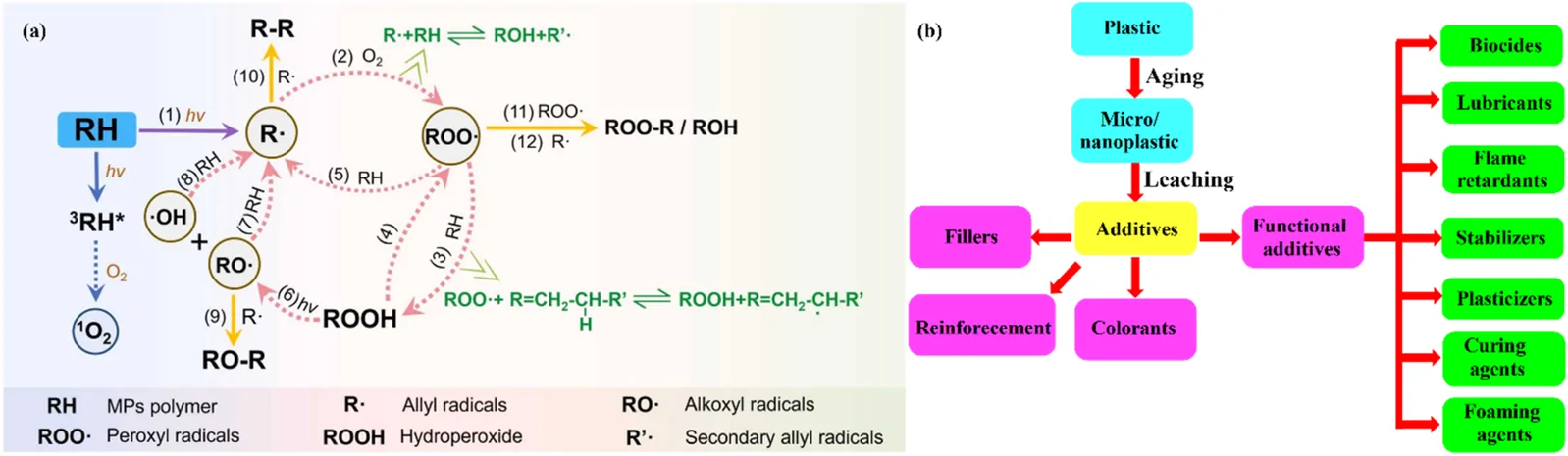
(a) Photoaging routes of plastics demonstrating the initiation, propagation and termination steps. Figure adapted from ref. 37 with permission from Elsevier, copyright 2025 under License Number: 6340650311164. (b) Leaching of additives from plastic in the aquatic environment to produce different products.

### 5.2 Leaching of additives

Plastic additives are purposely introduced during the manufacturing process to decrease the cost of the products and improve the service life and performance of plastic. These commonly include flame retardants, plasticizers and antioxidants. However, leaching of these additives causes significant environmental issues. Physical contact, metabolic activities, and ingestion may cause the leaching of additives from plastic into organisms. When plastic is ingested, it presents two potential hazards: first, it physically harms the animal, and secondly, it exposes the animal to hazardous chemicals leached from plastic during digestion. Since the additives are not chemically attached to the plastic, they escape easily. After their leaching, they may degrade to form secondary toxic pollutants that persist and accumulate in the environment. The leaching process is affected by various physical and chemical factors, such as additive size, solubility and volatility, temperature, permeability of the polymer matrix, and pH. In the natural environment, plastic undergoes a dynamic chain of physiochemical and biological transformations to form micro/nanoplastics. Sunlight results in the formation of reactive oxygen species to accelerate the leaching and degradation process to liberate various chemicals, as shown in Fig. 4b. The generated reactive species may degrade plastic and accelerate aging, which consequently results in the release and leaching of additives into the environment.^40,41^

## 6 Effect of micro/nanoplastics

### 6.1 Effect of micro/nanoplastics on animals and plants

Recently, the accumulation of microplastics in water, soil, and even the bodies of animals, plants and microorganisms has presented a new class of toxic materials with significant concerns. The presence of micro/nanoplastics affects marine life in two ways: first, when marine animals take in micro/nanoplastics, their internal organs and systems are affected directly, causing delayed growth, damaged immunity, blocked blood vessels and false satiety. Secondly, due to their excellent hydrophobicity and large specific surface areas, micro/nanoplastics easily adsorb hazardous chemicals and heavy metals to further toxify marine animals. It has been estimated that each person in the world takes in microplastics equivalent to 50 plastic bags per year, which is equal to one credit card, or 4.1 µg, per week for adults.^42,43^ Microplastics seem to interfere with the physiological and antioxidant enzymatic pathways in animals and plants to accelerate damage to cells and tissues, obstructing the two-way traffic of substances across the cell membrane. This results in the accumulation of toxic nitrogenous substances in the cells with decreased availability of essential elements and enzymes to the cell for major functions. Microplastics also accelerate the production of reactive oxygen species in marine plants and microorganisms, leading to significant oxidative stress. The kinetics of photosynthesis are significantly altered by inhibition of light absorption, CO_2_ capture and water availability, as these are the primary requirements for the production of glucose and oxygen. The microplastic-related risks to human beings are very serious, causing cardiovascular issues with remarkable adverse clinical consequences. Micro/nanoplastics have been progressively identified in human cardiovascular tissues such as blood and myocardium, demonstrating a systemic distribution and potential involvement of plastics in cardiovascular diseases. Micro/nanoplastics cause mitochondrial dysfunction, oxidative stress, inflammation, and even cell death of cardiac tissues, threatening myocardial damage, fibrosis and maladaptive remodelling. Further, the detection techniques are limited in sensitivity and standardization, resulting in under-reporting. Therefore, harmonized detection protocols and improved biomonitoring should be developed to deal with these issues.^44^ Numerous studies have investigated the effect of micro/nanoplastics on children. Pediatric populations are particularly exposed to environmental micro/nanoplastics pollution due to their early developmental stages and juvenile physiological defensive mechanisms. The presence of micro/nanoplastics has been confirmed in several organs and body fluids of children, with the lungs being recognized as the primary deposition place. Further, transplacental passage of micro/nanoplastics underscores fetal susceptibility to micro/nanoplastics. Similarly, the lower respiratory tract in children is the ideal place for micro/nanoplastics. Micro/nanoplastics may also disturb thyroid function in early life stages.^45^ The presence of microplastics in the human placenta and breast milk significantly affects hormonal activities. The detection of microplastics in testes and eggs increases infertility in males and females.^46,47^ Tiny plastic particles interrupt the synthesis, metabolism, and attachment of endogenous hormones, resulting in neurotoxicity. These abnormal hormonal activities disturb the function of the reproductive endocrine system. As a result, female reproductive tissues are damaged, which contributes to marked infertility. The mechanism does not operate in isolation. Rather, an interconnected pathological network exists leading to infertility. The tissues damaged by micro/nanoplastics may lead to cryptic ovarian autoantigens and autoimmune oophoritis. Autoimmune syndrome may impair ovarian function, resulting in early ovarian failure, which acts as a crucial pathogenic factor in female infertility. Mechanistically, micro/nanoplastics may impair the primordial follicle pool by initiating apoptosis and pyroptosis in ovarian cells, which is a key pathological factor driving female infertility.^48^ Microplastics have been detected in more than 1300 species, from invertebrates to vertebrates, demonstrating their effects on every sphere of life. Micro/nanoplastics are detected in the food we eat daily, the drinks we consume and even in the air we breathe.^49^ Microwave ovens are a major source of the induction of micro/nanoplastics in food items from plastic bowls. During microwave heating, it is estimated that more than 4.20 million particles are detached from plastic bowls with an area of 1 cm^2^ in only 3 min. Recyclable bottles have been found to contribute a considerable amount of micro/nanoplastics due to the wear and tear generated by their repeated use. Similarly, physical rubbing and friction of household items also add micro/nanoplastics to the environment.^50^ Micro/nanoplastics enter the human body through the skin, eyes, ears, nose, and mouth, as shown in Fig. 5.^51^

**Fig. 5:**
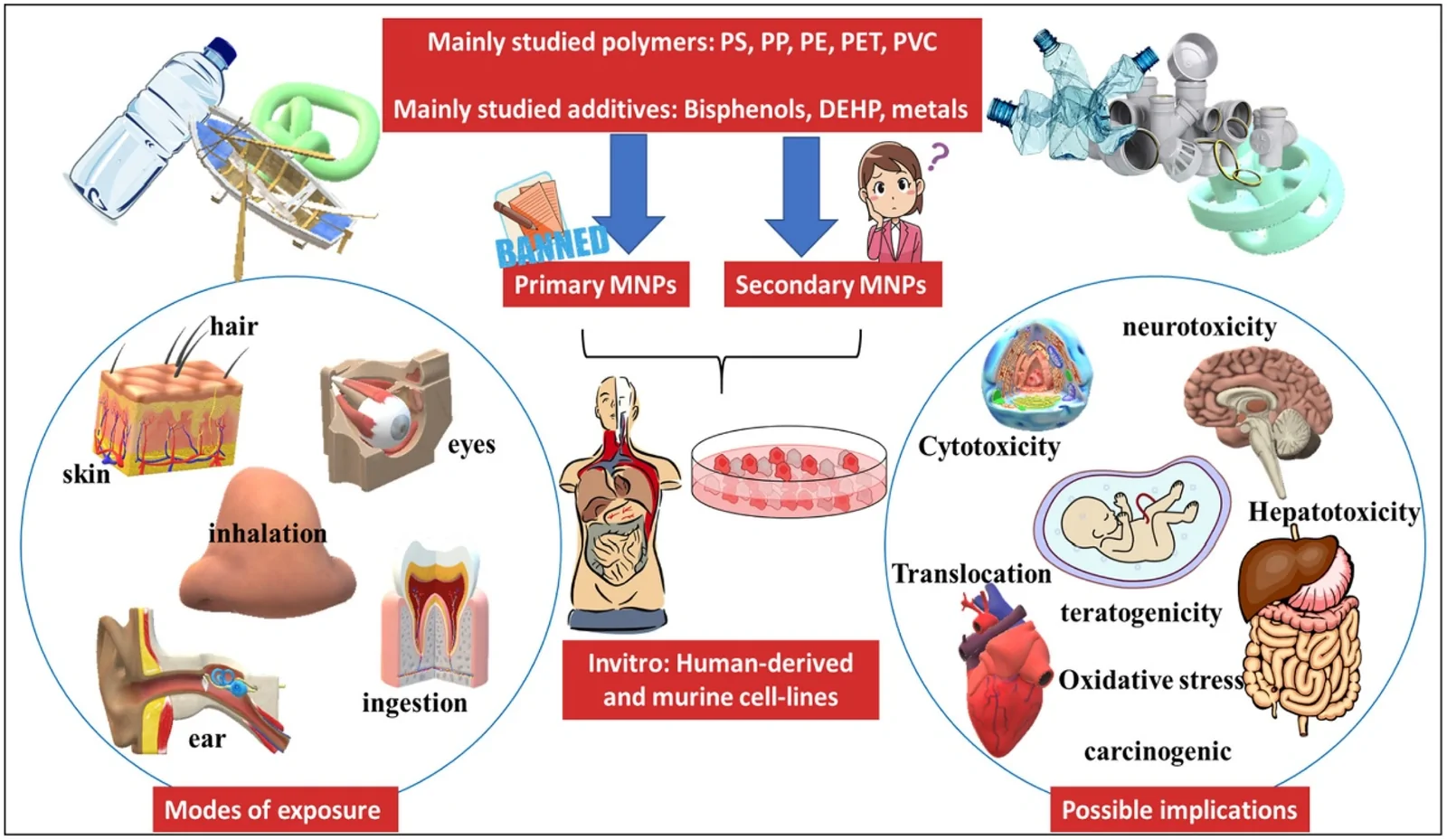
Different modes of ingestion of microplastics by human beings and their hazardous effects. Figure adapted from ref. 51 with permission from Elsevier, copyright 2025 under License Number: 6340650523027.

Several studies have indicated that the accumulation of micro/nanoplastics stimulates various types of human tumors. The formation of tumors is a multistep process caused by genetic, epigenetic, and environmental variations. Comparative studies have revealed a higher concentration of micro/nanoplastics in tumor samples, transforming normal tissues into malignant phenotypes. Cells incorporate micro/nanoplastics through passive membrane penetration, macropinocytosis, clathrin/caveolae-facilitated endocytosis, and even paracellular diffusion, causing severe disruption to cellular homeostasis. These highly stimulate multiple signaling pathways, damaging several organelles and ultimately accelerating cancer initiation and evolution. Continuous oxidative stress triggers receptor tyrosine kinases to stimulate mutagenesis, cancer development and progression. The micro/nanoplastics stimulate mitochondrial depolarization and augment reactive oxygen species accumulation to disturb mitochondrial respiration. These are classical indicators of oxidative-stress-motivated carcinogenesis. Micro/nanoplastics also stimulate DNA mutation, resulting in tumor initiation and progression. Chronic inflammation due to accumulated micro/nanoplastics is another factor in the progression of carcinogenesis.^52^ The presence of micro/nanoplastics in food, water and air results in ecotoxicological problems. They may cause alteration in gene expression, decrease fertilization rates, increase damage due to oxidative stress, and result in liver abnormalities in water animals such as oysters, zebrafish, mollusks, rainbow trout, and mussels. The toxic effects of micro/nanoplastics have been confirmed in mice, chickens, sea birds, cats, and dogs. In wheat plants, corn plants, oats plants, and tobacco plants, micro/nanoplastics cause low biomass, limited germination rates, and reduced growth. Micro/nanoplastics also act as a vector to adsorb toxic organic compounds, trace metals, and harmful additives at a rate that is multiple times larger than natural sediment.^53^ The stomach cells of many aquatic animals including fishes, frogs, turtles, and crustaceans taken from urban areas have been detected to possess higher amounts of microplastics in the form of hard fibers and small pieces, underlining the role of human activity. In animals and human beings, microplastics enter through skin contact, inhalation and digestion and assimilation.^54^ Airborne microplastics instigated from dust and storms enter the lungs through inhalation. Microplastics accumulated in plants, soil and edible fruits are taken up directly or indirectly through digestion and assimilation, and the absorption of microplastics through skin contact can be initiated when personal care items are used. After entering the body, the microplastics remain there for a long time, causing mechanical injury and inflammatory issues.^55^ Intestinal permeability increases, resulting in leaky gut condition, allowing toxic compounds to enter the bloodstream. The interaction of microplastics with biomolecules in the cell results in the precipitation and agglomeration of toxic materials in the cell, causing fatal diseases.^56^ To evaluate environmental and food safety hazards, Vleeming *et al.* examined the localisation and translocation of polystyrene nanoplastics in edible crops such as lettuce, carrot, and wheat.^57^ With the aid of confocal and scanning electron microscopy, clear internalisation of plastic nanoparticles was detected in the tissues of the leaves, roots, and stems of all three plants, with substantial differences among the selected species. Plastic particles were detected in the cortical and vascular tissues of the roots of all plants, as shown in Fig. 6a and b. A significant concentration of plastic nanoparticles was detected in the leaves (mesophyll tissue) and stem (xylem) of the plants, with slight variations in the upward and downward movement observed in all the plants, as shown in Fig. 6c and d. Interestingly, surface characteristics such as the trichomes, wax layer and stomatal densities of the plants substantially altered the presence and retention of plastic nanoparticles. The SEM and confocal images of the roots of lettuce and wheat shown in Fig. 7 demonstrate the adherence of plastic nanoparticles to the inner surface of the vascular bundle and cortical regions.

**Fig. 6:**
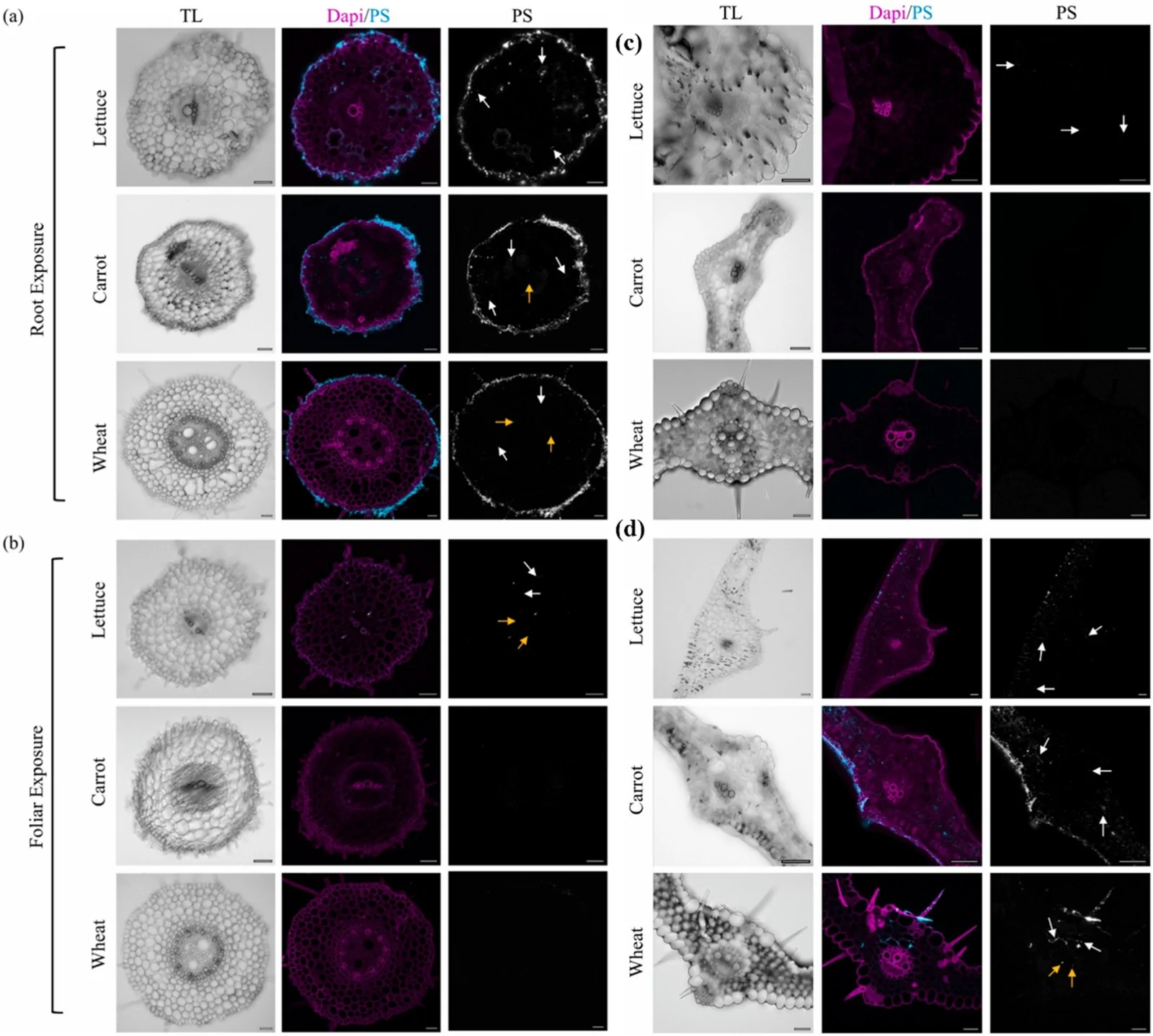
Images of the roots (lettuce, carrot, and wheat) following root exposure (a) and foliar exposure (b). Images of the leaf (lettuce, carrot, and wheat) following root exposure (c) and foliar exposure (d). Orange and white arrows indicate plastic nanoparticles found in vascular and non-vascular structures, respectively. Figure adapted from ref. 57 which is open access and allows the use of Figures under the Creative Commons CC BY 4.0.

**Fig. 7:**
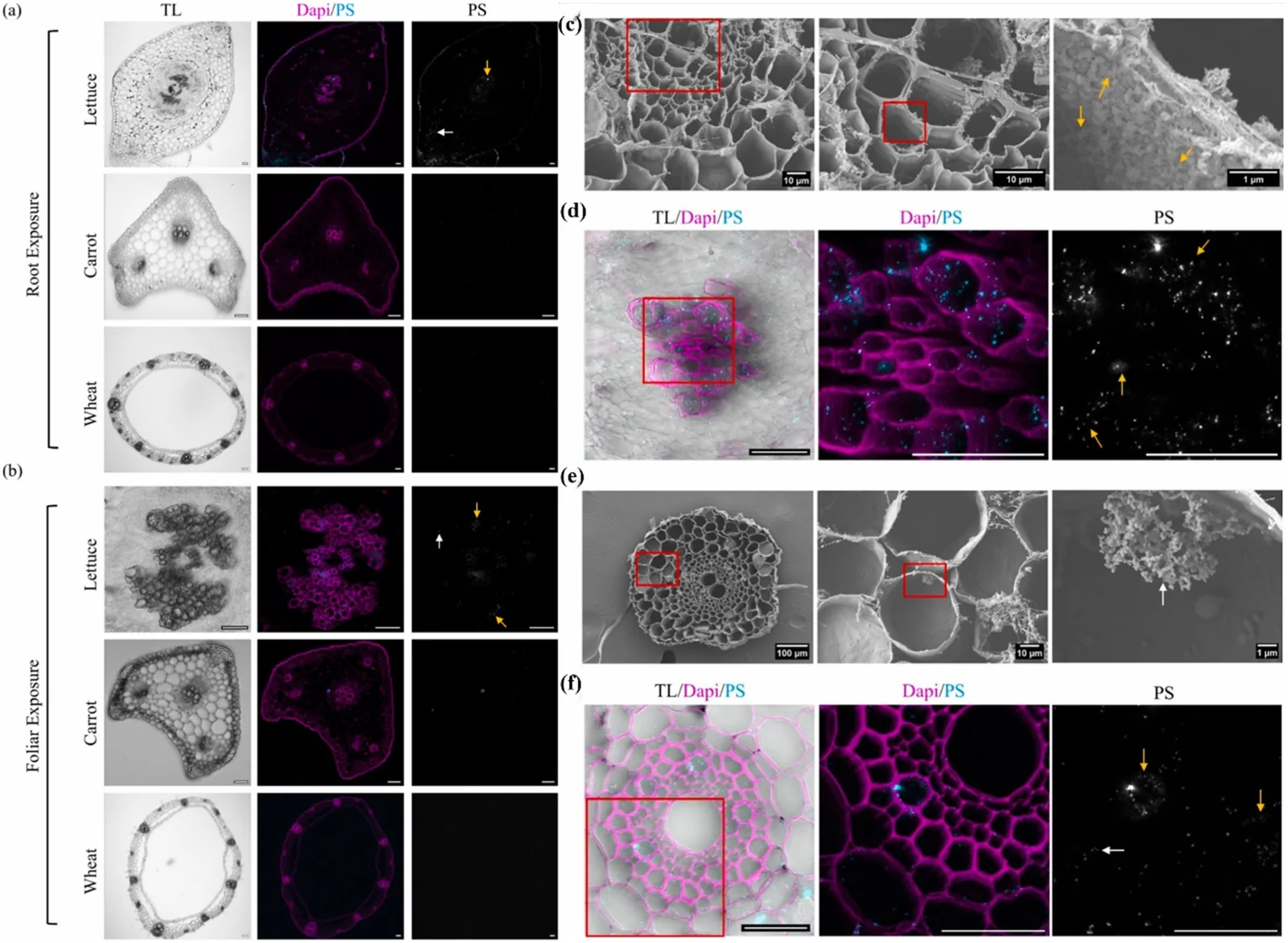
Images of the stem (lettuce, carrot, and wheat) following root exposure (a) and foliar exposure (b). SEM images of the roots of lettuce (c) and confocal images of roots with nanoparticles adhered to the inner surface of the vascular bundle (d). SEM images of the roots of wheat (e) and confocal image with plastic nanoparticles in the cortical region (f). Orange and white arrows indicate plastic nanoparticles found in vascular and parenchyma or cortical parts, respectively. Figure adapted from ref. 57 which is open access and allows the use of Figures under the Creative Commons CC BY 4.0.

### 6.2 Effect of micro/nanoplastics on soil and crop productivity

Soil is composed of agglomerates of small particles with a diameter of less than 10 mm. It is formed by cementation, combination, and clustering of soil particles. These aggregates play an essential role in normalizing the retention, holding and transportation of water, minerals and heat. The stability of soil aggregates is an important parameter of soil fertility and health. The aggregates of soil support the organic carbon in the soil and significantly improve the ability of soil to retain carbon sequestration.^58^ Microplastic pollution is directly associated with changes in the structure, fertility, and bio-activity of soil, with significant consequences on soil functionality, crop growth and crop productivity, and food security. Irrigation certainly accelerates the vertical migration of microplastics in soil. Some indoor activities are responsible for the downward migration of microplastic particles through soil cracks and cavities. This transport and migration of microplastics is altered by several factors, including the size of the microplastic particles, the salinity of the soil, holes and cracks in the soil, and the water penetration and capture capabilities of the soil.^59^ Generally, large plastic particles remain on the surface, while small particles penetrate deep into the soil. It is estimated that the soil organic carbons are significantly altered by many factors including moisture, microbial colonization and pH. However, the effect of microplastics is drastic. The gradual accumulation of micro/nanoplastics in soil is reported to be a considerable risk to soil organic carbons and soil aggregation. Plastic and its derivatives greatly affect the physiochemical behavior and character of soil by modulating bacterial diversity and water retention, and hence, crop production and plant growth. The special ability of micro/nanoplastics to adsorb heavy metals and toxic materials owing to their large surface areas plays a significant role in the migration and transportation of toxins, spreading pollution-based diseases to all parts of the soil.^60^ Micro/nanoplastics can negatively disturb the physiochemical stability of soil aggregates and organic carbon. The ability of microaggregates to form macroaggregates is restricted by microplastics through the establishment of fractures and cracks. Once the soil aggregates are fractured due to the arrival of microplastics, the captured organic carbons are released. This greatly accelerates the degradation and mineralization of soil organics. Microplastics that are easily biodegradable essentially retard crop production by rapidly mineralizing essential elements and organic matter required for the gradual growth and health of plants.^61^ The high surface area and modulated hydrophobicity of micro/nanoplastics cause them to act as carriers for toxins, thus affecting their bioaccumulation and environmental destination. The toxicity of heavy metals is not only measured in terms of their nature, but also their chemical states and forms, which exhibit different environmental and biological activities under different circumstances. It is estimated that the content of heavy metals, especially acid-soluble metals, is significantly high in soil containing microplastics. The adsorption and desorption of Cd are decreased and increased, respectively, in microplastic-polluted soil.^62^ Micro/nanoplastics impose a suppressive effect on the nitrification and denitrification of soil. They alter and lower the amount of available nitrogen by adsorbing nitrogen compounds. Since nitrification transfers NH_4_^+^ to NO_2_^−^ and NO_3_^−^, micro/nanoplastics may cause a decrease in nitrogen in soil *via* liberating N_2_O. N_2_O has a global warming capability 300 times greater than CO_2_ and also accelerates the depletion of ozone layer, as shown in Fig. 8.^63^

**Fig. 8:**
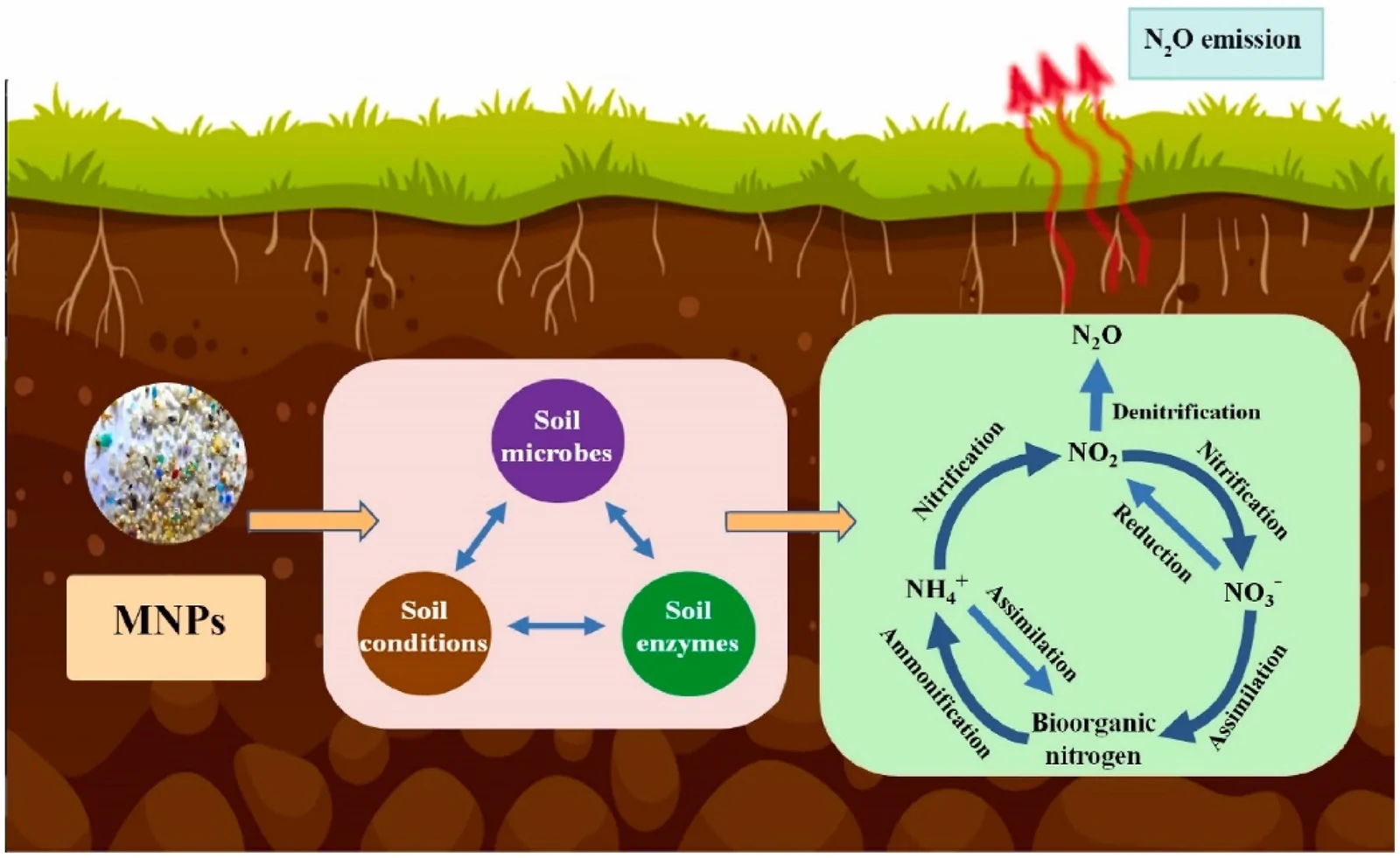
Effects of plastic on soil nitrogen and N_2_O release. Figure adapted from ref. 63 with permission from Elsevier, copyright 2025 under License Number: 6340651261696.

## 7 Recycling of plastics

Plastic recycling is crucial for the conservation of the natural environment, as it significantly reduces dependence on the fossil fuels and petrochemicals required for the synthesis of plastic goods. Therefore, the accumulation of plastic and its derivatives in the environment, plants and animals has led to colossal research activity globally. Amongst plastics, thermoplastics are a type of moldable plastic and can be recycled using various chemical and biological techniques. These include polyethylene terephthalate, low-density polyethylene, polystyrene and polypropylene.^64^

Several techniques have been followed to monitor and reduce the impacts of plastics, especially microplastics and nanoplastics. The recycling of plastic is generally classified as primary recycling processes, secondary recycling processes and tertiary recycling processes. In a primary recycling or closed-loop recycling process, the original pristine post-consumer waste plastic of a known source is used to form polymeric materials, such as the synthesis of plastic bottles from blends of reprocessed polyethylene terephthalate (PET). Secondary recycling processes are the most used techniques, in which the processed plastic is primarily used for purposes other than that for which the original polymer was synthesised. The secondary recycling process offers products that are inferior in value to the waste plastic, and are usually termed as ‘downgraded’ or ‘downcycled’ products.^65^ Both primary and secondary recycling processes are mechanical in nature; the waste plastic materials are separated, crushed, cleaned and extruded into new products. The tertiary recycling process is based on the chemical treatment of plastic wastes and is generally employed for the recovery of the original monomers (petrochemical components).^66^ Both chemical and mechanical procedures have been employed to remove plastic and its derivatives from the environment. It has been estimated that only 70% of the plastic used globally is recovered through chemical and mechanical recycling, while the rest is discharged to the external environment. In the following section, we discuss both chemical and mechanical treatments of waste plastics to recycle them for reliable usage and application.^67^ Just as pollution is a one-way trip, engineered plastics and their constituents move in a linear economy. The adverse environmental effects of plastics have pushed the world to move from a linear economy to a circular economy by recycling various plastic wastes into their original monomers, fuels or new products, as shown in Fig. 9.

**Fig. 9:**
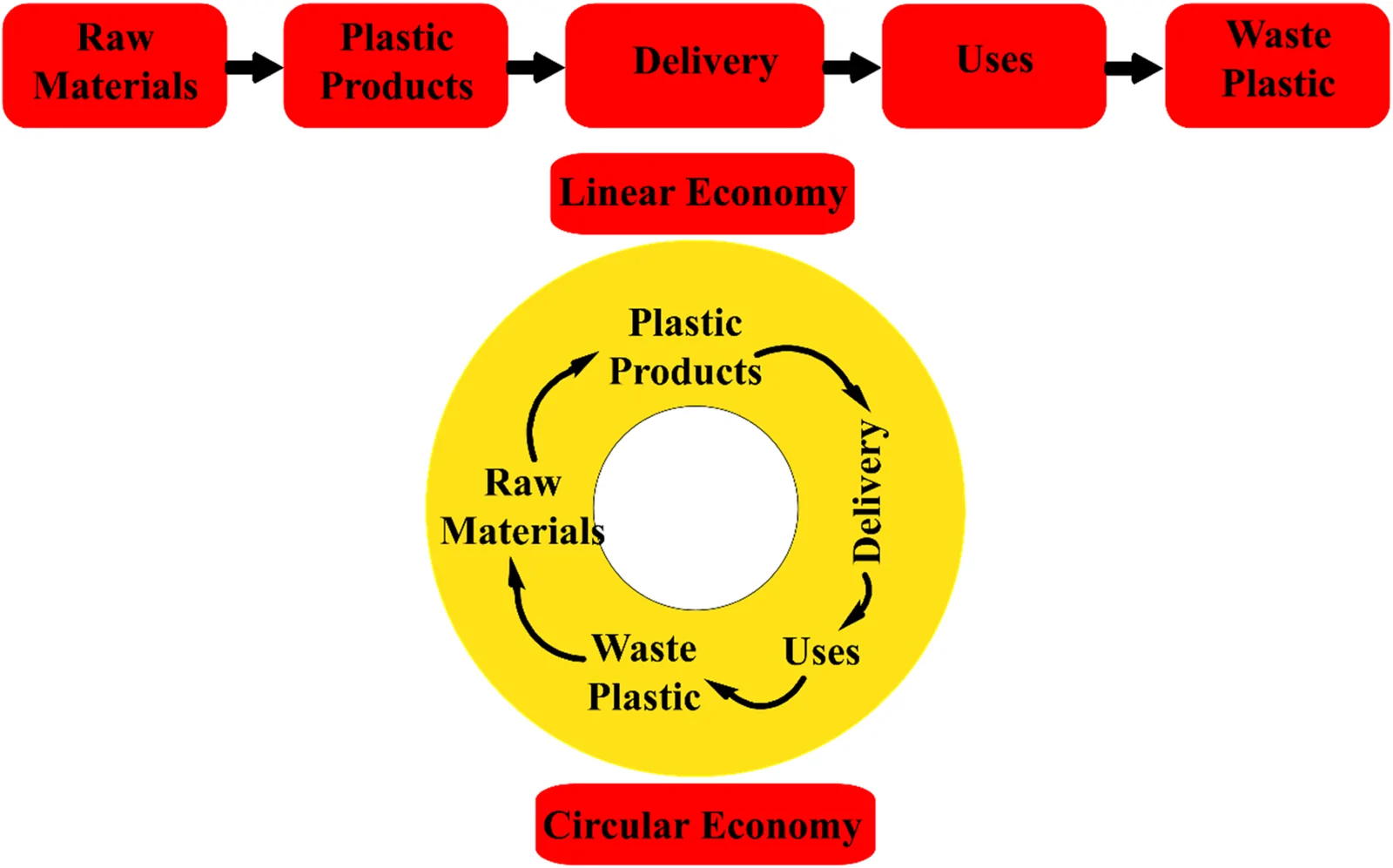
Graphical representation of linear and circular economies.

### 7.1 Chemical recycling

Chemical recycling, which is also known as depolymerization, advanced recycling or molecular recycling, is a very promising technique, as it preserves the value and potential of the post-prepared polymers by translating the raw plastic into its original starting materials, fuels or value-added chemicals. The process has been developed as a potential area of research in chemical and engineering industries. The major aim of chemical recycling is the selective conversion of plastic wastes into isolable and value-added chemicals. The polymers are treated chemically to break the long chemical bonds between the constituent elements to form monomers that are ready for preparation in the next phase. The chemical recycling path is generally achieved either thermochemically or catalytically. An example of chemical recycling is the pyrolytic conversion of waste polymer into its pure monomers under high-temperature conditions in the presence of a suitable catalyst. In this process, the saturated hydrocarbon skeleton is first functionalized, followed by its depolymerization to form the original monomers. For the rapid and selective conversion of the polymer, supercritical liquids (water, *etc.*) are generally used as suitable reaction media and solvents for the decomposition to depolymerize and solvolyze the condensation polymers into the original monomers without using catalysts. The supercritical liquids are also used to depolymerize addition polymers into their original monomers. Achieving the chemical recycling process under mild conditions has motivated huge amounts of research. The process is a complex procedure, and requires enormous investment and setup of infrastructure. The process is also not cost effective, as it requires additional chemicals and energy-intensive consumption to achieve high efficiency. Therefore, large-scale implementation of chemical recycling is still an issue, and thus, more efficient ways to treat waste plastics are under consideration.^68^

### 7.2 Mechanical recycling

Mechanical recycling is the most cost-effective technique, typically resulting in re-granulated materials. It offers recovery of wastes through mechanical methods (secondary raw materials), processing of the collected wastes and their conversion into new commodities without modifying the chemical assembly of the polymer. The process includes sorting and separation, grinding and crushing, washing and cleaning, and re-granulation and granulation of the waste materials to be used in the recycling process. The process preserves the natural environment and reduces the energy consumption needed to form the virgin materials. However, the process contributes a huge quantity of greenhouse gases, as the melting and processing of the waste materials require electricity and hydrocarbon fuels. Huge amounts of volatile organic materials are liberated into the atmosphere. Removal of these volatile compounds reduces hydrolysis and acidolysis and enhances polymer melt odour to improve the value of the recyclates. The products formed through mechanical recycling are inferior in quality, as the separation of plastic materials of different natures is not complete, and the mixture of different plastics leads to the formation of low-quality recycled products. A combination of both chemical and physical forces significantly affects the tensile strength and elongation of the recycled products. To account for this, recycling industries use an “open-loop” or “semi-closed-loop” system in which virgin plastic is mixed with recyclable materials. Additionally, the products cannot be re-processed again. Ultimately, the recycled plastic is discharged to the external environment as landfill. However, by controlling the process, its challenges and parameters, the process may be improved into a sustainable practice.^69^ Both the chemical and mechanical recycling of plastics are shown in Fig. 10.

**Fig. 10:**
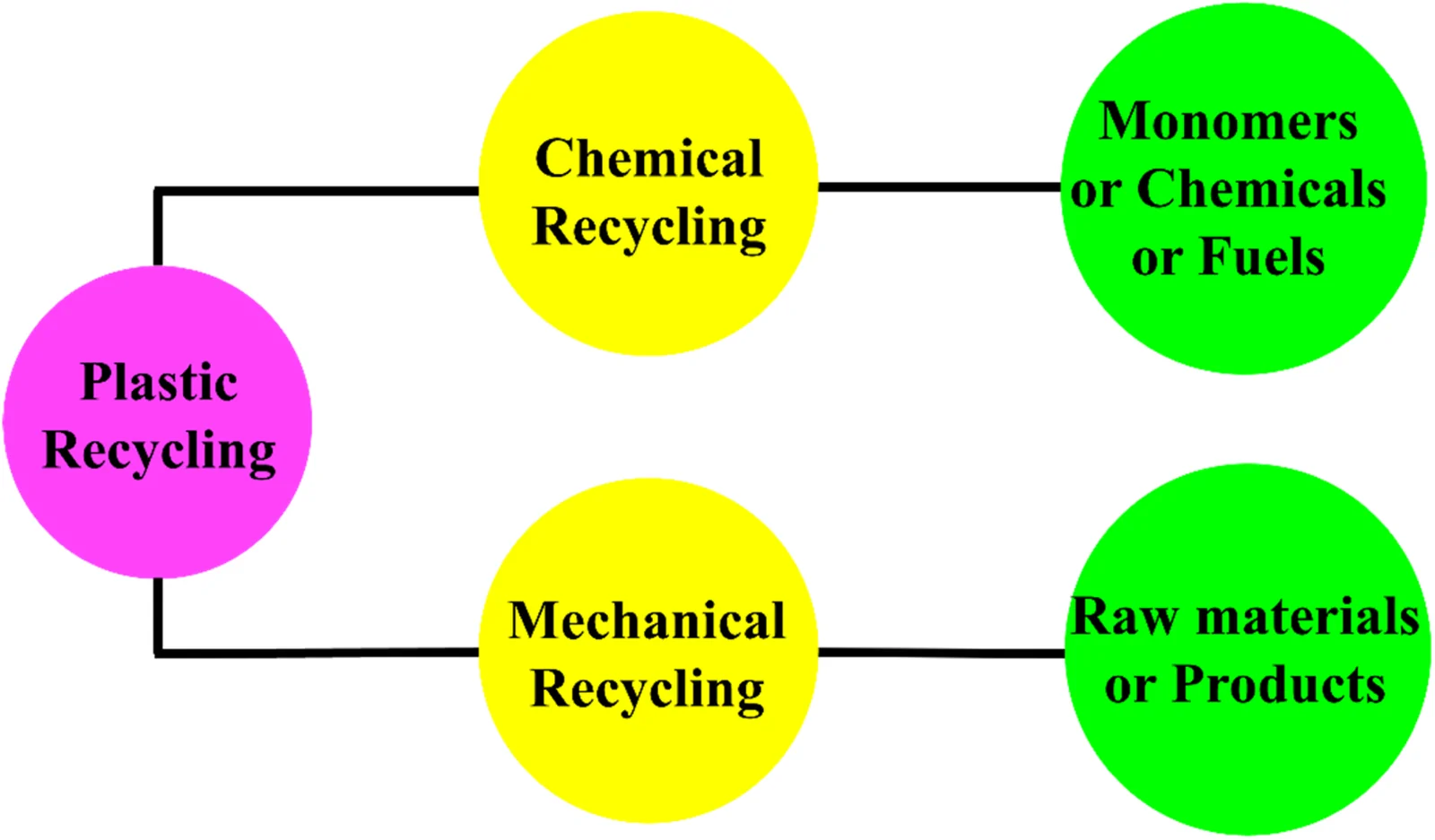
Chemical and mechanical recycling of plastics.

## 8 Plastic eradication methods

The separation and removal of microplastics and nanoplastics from the environment is a tedious process for which various techniques have been attempted and tested. Great efforts have been devoted to finding efficient handling techniques. Due to the grave flaws of traditional wastewater treatment techniques for the removal of organic and inorganic pollutants, it is crucial to work out new and advanced sustainable eradication methods for the removal of microplastics and nanoplastics. It is highly imperative to capture minute-sized plastic particles with variable forms and chemical composition.^70^ Currently, various biological, physical, and chemical techniques are in use to deal with plastic and its environmental hazards. Biological methods include biofiltration or biochar filtration, and bioreactor and dynamic membrane separation. Physical techniques involve filtration, magnetic separation, density separation, floatation, oil film formation and adsorption, while the chemical procedures comprise coagulation, flocculation, ozonation, electrochemical eradication, and photocatalytic oxidation. Biological techniques include biodegradation. In the following sections, we have discussed some of these eradication techniques in detail along with the removal mechanisms.

### 8.1 Biological techniques for the removal of microplastics and nanoplastics

Plastic-based environmental pollution is no longer an isolated matter. It is not an individual issue, but a global issue that requires multi-scalar and interdisciplinary works. The recognition of microplastics/nanoplastics as emergent contaminants underscores the imperative need for their removal to protect and safeguard the environment. To preserve biodiversity and ecological stability, microplastics/nanoplastics should not be allowed to penetrate the food chain. A massive reduction in plastic synthesis and consumption must be accomplished through the generation of innovative biodegradable products. As for existent plastics and their derivatives, their removal is of prime importance to restrict their accumulation on land, in the ocean and in the atmosphere.^71^

Biological techniques are cost-effective and sustainable approaches for the elimination of micro/nanoplastics with the aid of plants, microorganisms (bacteria, actinomycetes, algae, and fungi) and enzymes. They are simple, green, and energy efficient procedures that have long existed. In these processes, plants and microorganisms consume plastic as a source of carbon and nitrogen for their growth and multiplication. The biotic removal of micro/nanoplastics is an exceptional substitute for conventional chemical and thermal removal techniques, particularly when used as an integrated and sustainable circular economic model. This process proceeds through the secretion of enzymes by microorganisms, which work as a tool for the disintegration of large polymeric chains into small monomers, followed by their conversion into CO_2_ and H_2_O.^72,73^

The biotic removal of micro/nanoplastics is a complex procedure facilitated by plants and microorganisms frequently found in a bacterial community (complex consortia).^74–76^ In phytoremediation, in which plants are grown on land tainted with plastics, micro/nanoplastics with different mechanical properties are taken up by plants. The micro/nanoplastics are frequently found in the roots, leaves and even seeds of such plants. To restrict the accumulation of micro/nanoplastics to only root nodules, certain plants are functionalized to reduce the transport of plastic particles through the secretion of certain organic acids. As a result, the accumulation of micro/nanoplastics in the seeds and fruits is prevented, and the accumulated plastic particles are degraded into simple non-toxic compounds inside the root nodules. The phytoremediation of plastics significantly depends on the plant species, soil type, and other environmental conditions.^77–79^

In case of microbial (algal) removal of plastics, the elimination mechanism is more complex and involves several steps. Each stage of the elimination mechanism is controlled by both external factors such as pH and temperature, and internal factors such as the surface chemistry, crystallinity and hydrophobicity of the plastic materials. Huge amounts of research have been focused on developing bacterial variants with exceptional substrate adherence, enhanced thermal (high-temperature) stability, and improved removal efficiency through chemical engineering. Microbial removal is initiated with the adhesion or biosorption and colonization of microorganisms on the surface of plastic. The preferred spots of attachment for the microorganisms are certain functional groups (OH, carbonyl, and carboxylic, sulfonate) present on algal cells. These effectively interact with microplastics through mechanisms such as biosorption and surface adhesion.^80^ The process can be significantly accelerated using several strategies, such as UV irradiation and exposure to certain chemical compounds at high temperature, which significantly alter the crystallinity and tensile strength of the plastic.^81^ As a result of the hydrophilic nature of microplastics, microorganisms develop biofilms on the plastic surface through hydrophobic and electrostatic interactions. The adhesion of microorganisms is followed by their colonization, which helps to localize the microbial activity through the establishment of a conducive environment for the chemical reactions of the secreted enzymes.^82^ The growth of colonies and secretion of enzymes are altered by the nature of the plastic, pH and the temperature of the environment. Micro/nanoplastics with rough surfaces provide abundant active sites for the attachment of microorganisms, while lower hydrophobicity assists their colonization. Poor crystallinity ensures augmented bacterial diversity, while certain additives such as biocides and antifouling (chlorothalonil) compounds, as well as high salinity, lower the elimination of micro/nanoplastics. The mass of the microbial colony and its growth depend on the fate of accessible nutrients on the surface of plastics and in the nearby surroundings.^83^ The process of microbial colonization is not limited to land only, but also takes place in water, and the general concept of a ‘plastisphere’ is used. The microbial colonization is followed by the secretion of essential extracellular enzymes (PETase, MHETase, laccases, peroxidases, cutinases and esterases) to realize the depolymerization of the polymeric structure into small monomers. The secreted enzymes are highly selective and attack specific chemical bonds in the polymeric backbone for the degradation and depolymerization process.^84,85^ Polymers such as polyethylene and polypropylene, in which the backbones are primarily composed of hydrocarbons, are relatively resistant towards enzymatic action, while polymers with ester, ether, and amide functional groups are more vulnerable to enzymatic action. For example, polyethylene terephthalate is first hydrolysed by PETase into hydroxyethyl terephthalate, followed by its conversion into terephthalic acid (C_8_H_6_O_4_) and ethylene glycol (C_2_H_6_O_2_) with the aid of MHETase. Polymers whose backbones are composed of aromatic rings are degraded by enzymes such as laccases and peroxidases secreted by fungi and bacteria, while aliphatic-based polymers are destabilized by enzymes such as cutinases and esterases. In the final stage, the formed intermediates are mineralized into simple non-toxic CO_2_, CH_4_ and H_2_O through intracellular metabolism pathways. An aerobic environment promotes the formation of CO_2_ and H_2_O, while an anaerobic environment facilitates the formation of CO_2_ and CH_4_, as shown in Fig. 11. However, the real-world enzymatic applications are limited by the nature of plastic, external environment, cost, and a lack of universal enzymes to target a broad range of micro/nanoplastics.^43^

**Fig. 11:**
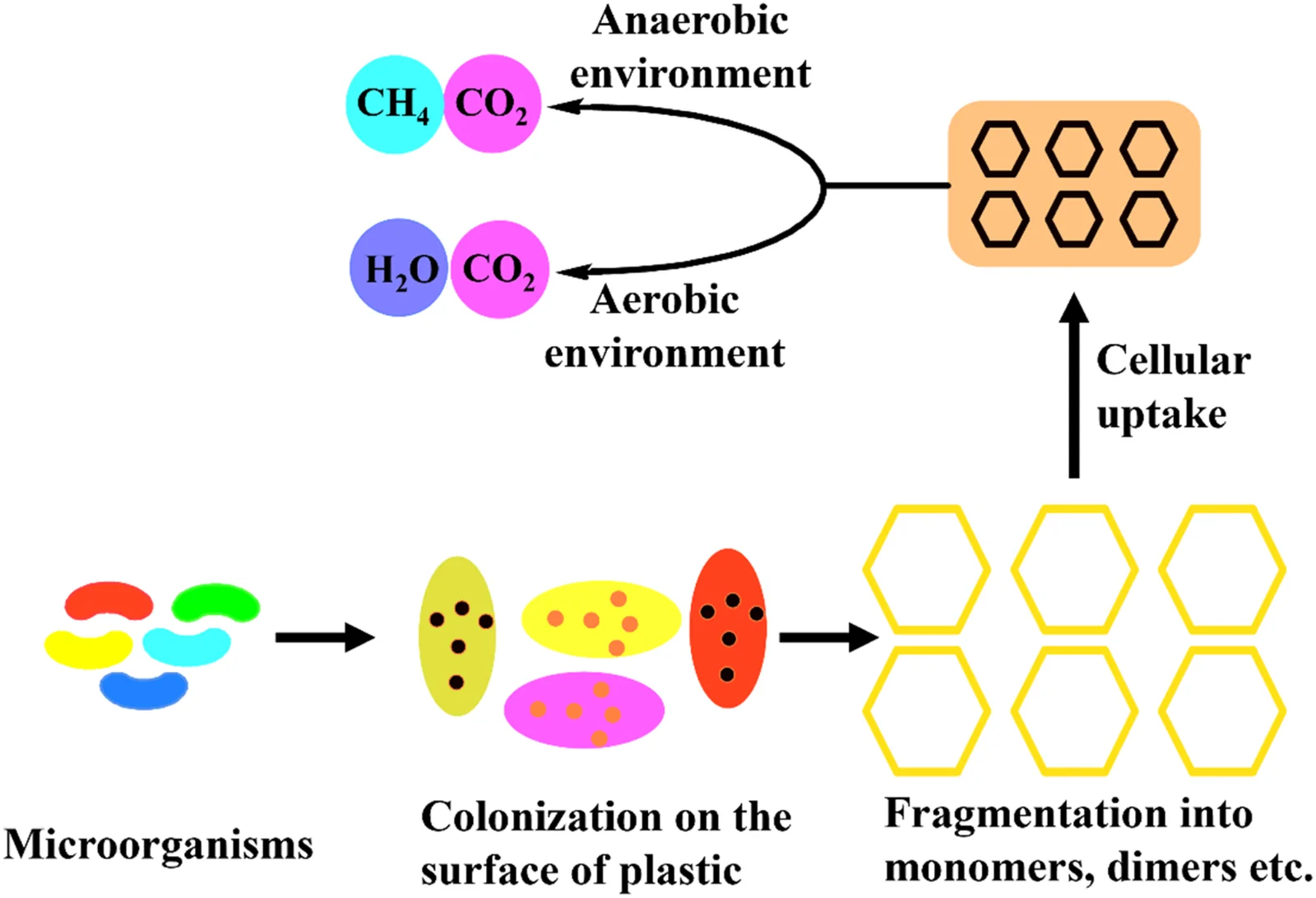
Schematic of the microbial degradation of micro/nanoplastics.

### 8.2 Physical techniques for the removal of microplastics and nanoplastics

#### 8.2.1 Adsorption

Adsorption is one of the most primitive and attractive techniques used to remove toxic materials of different types. It is a physico-chemical technique in which the particles of interest are allowed to adhere on the surface of a solid, which is known as the adsorbent. The process has the benefit of being relatively cheap and easy. However, the replacement of the exhausted adsorbent and regeneration of fresh adsorbent are tedious and costly.^86^ The choice of a suitable adsorbent to attract and adhere micro/nanoplastics of diverse natures is crucial in this regard. Generally, adsorbents with porous structures and large surface areas are very promising in the removal of micro/nanoplastics. Both synthetic and natural adsorbents have been efficiently used to remove micro/nanoplastics from water. Natural adsorbents are highly effective and have several remarkable benefits. Biochar, cellulose, woodchips, sawdust, algae and seaweed, corn straw, pineapple peels, sugarcane bagasse, artichoke leaves, and clay minerals such as bentonite, kaolinite, and montmorillonite *etc.* are amongst the commonly used natural adsorbents, while MOFs, activated carbon and graphene, and zeolites represent a cutting-edge class of synthetic adsorbents. The adsorbent selected for efficient eradication depends on the type of micro/nanoplastics, and their size, hydrophobicity and composition. Each parameter has its individual advantages.^87,88^ Efficiency and long-term stability and feasibility are critical. Regeneration and environmentally friendly disposal of used adsorbents to prevent secondary pollution (due to the leakage of adsorbents into the environment) are very important. Suitable reuse techniques (chemical, thermal and electrochemical) allow several adsorption–desorption rotations while maintaining the performance efficiency. For commercial applications, the adsorptive removal of pollutants and remediation of environment must combine cost, efficiency and sustainability. The commonly used incineration and landfilling techniques present numerous environmental issues, with substantial leaching of toxic materials. Workable solutions to renovate used adsorbents into suitable materials would enable a circular economy to reduce environmental impact.^89^ Some well-known physical techniques are summarized in Fig. 12.

**Fig. 12:**
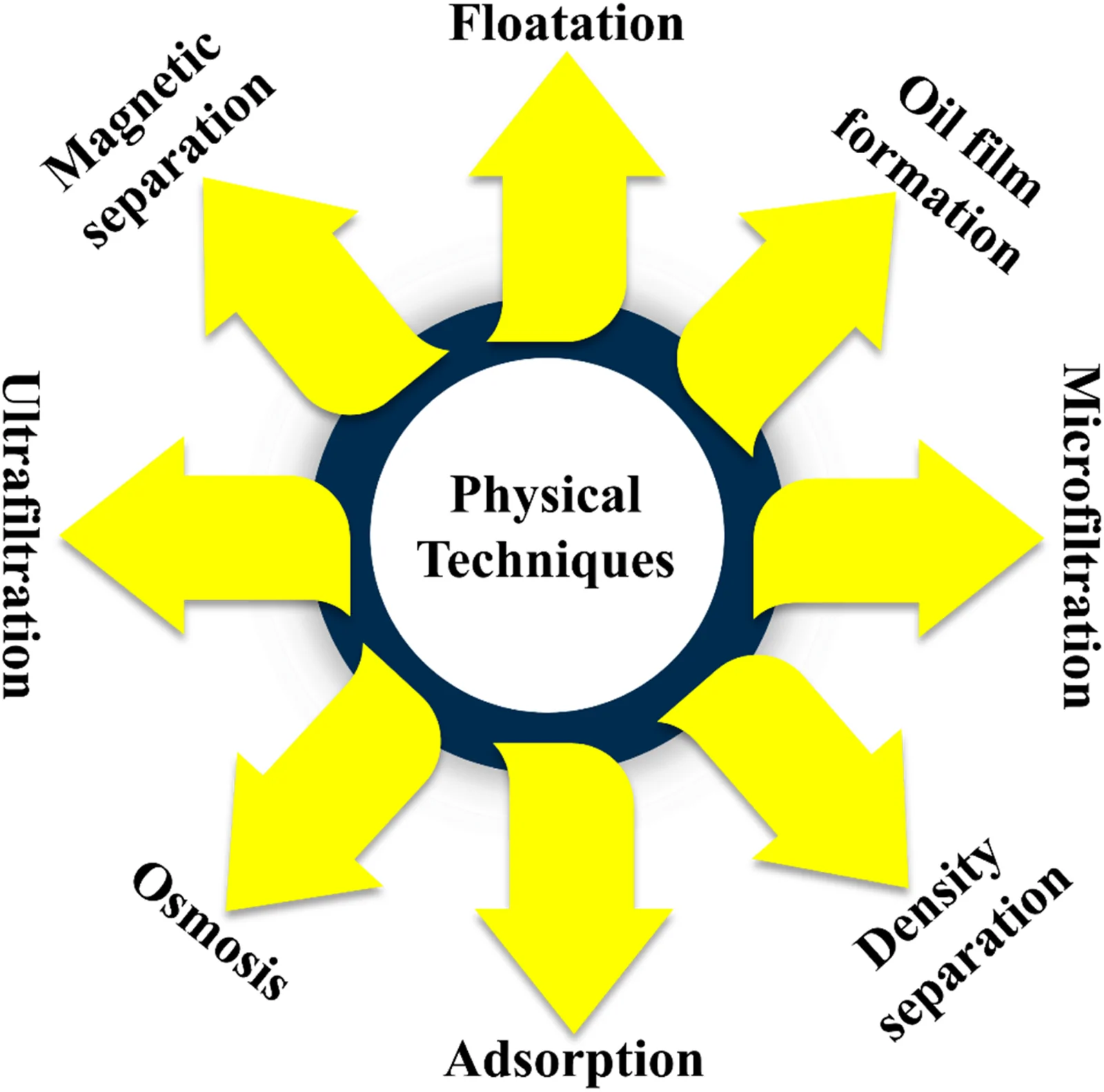
Physical techniques for the removal of micro/nanoplastics.

The adsorption of micro/nanoplastics on the surface of adsorbents is governed by several forces, such as hydrogen bonding, π–π pairing, capillary forces, van der Waals intermolecular interaction, electrostatic forces, hydrophobic–hydrophobic interaction and surface complexation. The involvement of these forces can be monitored through FTIR, XPS and other spectroscopic techniques. Generally, plastic materials contain significant amounts of C–C single bonds. When adsorbed on the surface of a suitable adsorbent, the XPS intensity of these bonds, generally at 284.82 eV, is augmented, demonstrating the binding of plastic to the surface of the adsorbent. The excellent hydrophobicity of plastic materials allows them to adsorb through hydrophobic forces and van der Waals forces and π–π and even *σ*–π pairing with the amino, hydroxy and carboxyl groups on the surfaces of natural adsorbents, while electrostatic forces, hydrogen interaction and π–π pairing play vital roles in the removal of plastic materials through synthetic adsorbents, as shown in Fig. 13a and b.^90–92^

**Fig. 13:**
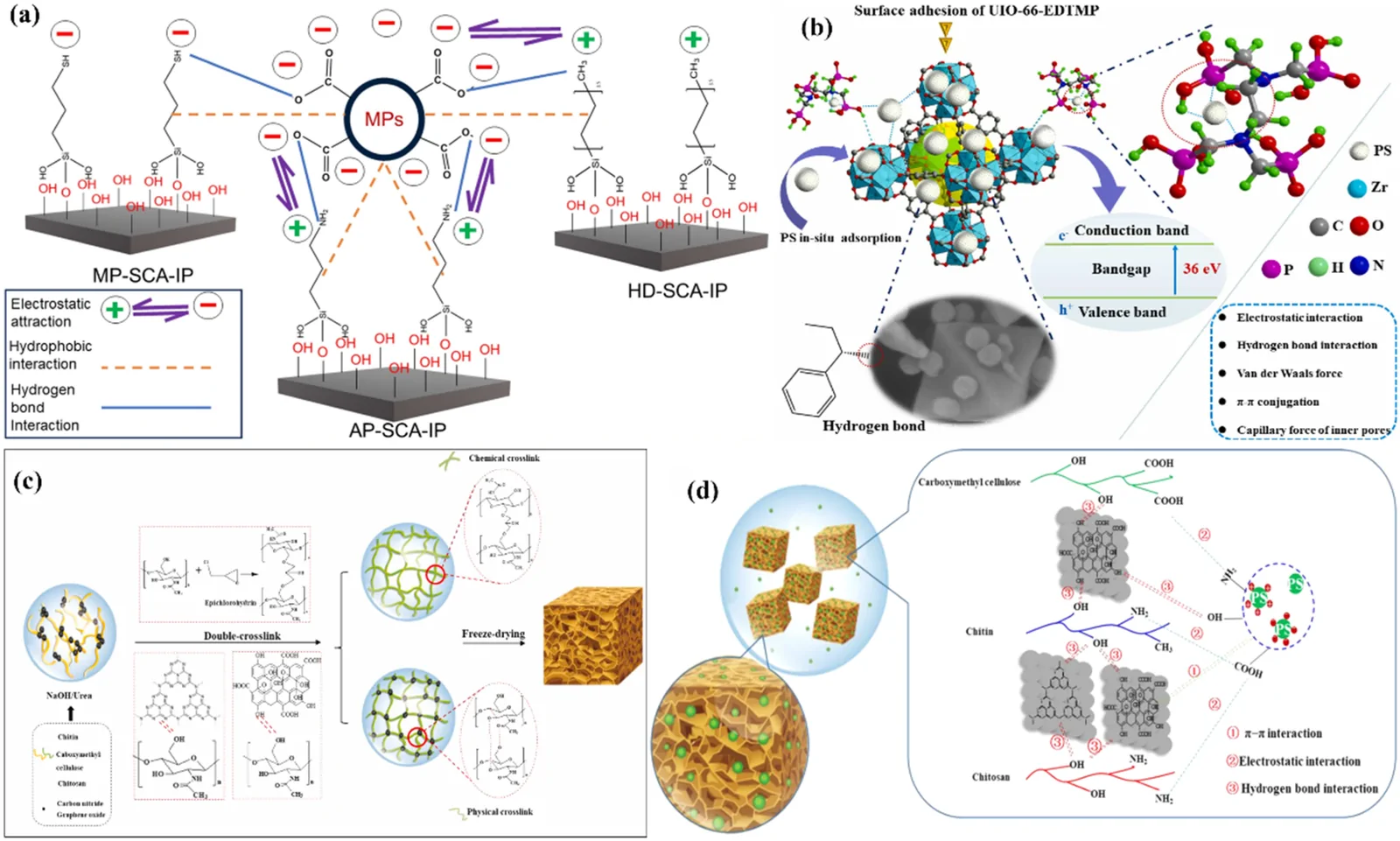
(a) Mechanism of the adsorption of microplastics on the surface of a modified natural adsorbent. Figure adapted from ref. 91 with permission from Elsevier, copyright 2025 under License Number: 6340651508121. (b) Mechanism of micro/nanoplastics adsorption on the surface of a typical synthetic adsorbent. Figure adapted from ref. 92 with permission from Elsevier, copyright 2025 under License Number: 6340670293875. (c) Synthesis of a chitin-based sponge, and (d) proposed mechanism for the adsorptive removal of micro/nanoplastics. Figure adapted from ref. 100 with permission from Elsevier, copyright 2025 under License Number: 6340670558294.

As low-cost materials, biochar-based adsorbents have attracted intensive research interest recently. Biochar is a solid carbonaceous material formed when biomass, agricultural wastes and municipal wastes are treated under low oxygen content at high temperature to remove volatile substances and enrich the carbon mass in the solid material.^93–95^ Biochar is a sustainable material having a porous structure with an excellent ability to bind and adsorb micro/nanoplastics from soil and water. The presence of multiple carbonaceous and oxygenated functional groups and certain important elements such as alkali and alkaline earth metals, along with nitrogen and sulphur, make biochar one of the most suitable adsorbents for the removal of plastic-based materials. The high preparation temperature results in a large surface area and exceptional stability to incorporate micro/nanoplastics from a variety of sources.^96^ The plastic removal efficiency of biochar depends on the polarity and aromaticity of the biochar and the physical and chemical structure of the plastic.^97^ Owing to their hydrophobic character and surface charges, micro/nanoplastics are easily attracted to biochar *via* surface complexation and electrostatic attraction. Since plastic is an excellent adsorbent for metals and organic pollutants in aqueous environments, the interaction of these metals and biochar provides a driving force for the removal of metals, pollutants and micro/nanoplastics.^98,99^ In addition to the adsorption mechanisms discussed earlier, some recent studies have also focused on improving the structure of adsorbents to enhance their micro/nanoplastic removal efficiencies. One effective approach is the development of crosslinked adsorbents such as chitin-based sponges. In this method, polymer chains are connected through chemical or physical bonds to form a stable three-dimensional network structure. For example, chitin-based sponges can be chemically crosslinked with epichlorohydrin and modified with graphene oxide, oxygen-doped carbon nitride or carboxymethyl cellulose.^100^ Similarly, starch–gelatin composites are synthesized by combining chemical and physical crosslinking, followed by freeze-drying to create porous structures.^101^ As shown in Fig. 13c and d, these crosslinked structures provide increased surface area, high mechanical stability, and a large number of functional groups. These characteristics support the adsorption of micro/nanoplastics; in particular, functional groups such as hydroxyl, amino, carboxyl help to improve the overall adsorption efficiency *via* interactions such as hydrogen bonding, electrostatic attraction and π–π interactions.

#### 8.2.2 Density separation

Separation on the basis of differences in the densities of plastics and other substances is a simple technique by which micro/nanoplastics are separated from water or sediments. The process is based on the principle that when substances with different densities are fed into a separating container having a liquid of a certain density, the heavier substance sinks to the bottom, while the lighter one floats to the surface.^102^ As a result of this sink-float separation, the micro/nanoplastics are separated efficiently. A water-based solution is generally used in the density separation procedure. This technique is primarily used to separate micro/nanoplastics from water, sands and sediments, as the density of plastic materials (0.8 to 1.4 g cm^−3^) is substantially very low compared to the density of sediments.^103,104^ Micro/nanoplastics are separated from water samples through a floating procedure. Factors controlling the density separation efficiency include the time required for the micro/nanoplastics to adhere and agglomerate with each other, the adherence of bubbles and drops to the micro/nanoplastics, and the hydroscopic resistance of micro/nanoplastics to falling. The kinetics of the density separation of micro/nanoplastics is a function of their buoyancy as monitored by differences in the density of the plastics and the separation medium, the viscosity of the medium and the hydrodynamic character of the micro/nanoplastics.^105,106^

The amenability of micro/nanoplastic particles to density separation depends on their minimum size, shape and morphology, and surface and hydroscopic properties. The maximum size of the micro/nanoplastic particles is generally determined by the limitations of the instruments used for separation and the degree of disintegration and agglomeration of particles. If the densities of the micro/nanoplastic particles are much closer, their separation is difficult. If the micro/nanoplastic particles are smaller and thinner, their surface-to-weight ratio will be high, making them more hydrophobic due to surface tension, which substantially decreases their density separation. This method has the drawback of using salt as a density separator, which may cause additional costs and secondary pollution. Since plastic materials adsorb many substances on their surfaces, which cause substantial variation in density, it is therefore difficult to separate them completely.^107^

#### 8.2.3 Ultrafiltration

Removal of micro/nanoplastics through membrane techniques (ultra/microfiltration) has received significant attention owing to its simplicity, effectiveness and cost.^108^ These processes work on the principle of a physical hindrance mechanism that selectively allows through some materials and blocks others using size-controlled pores. Amongst these procedures, ultrafiltration is the most attractive and efficient procedure to deal with micro/nanoplastic pollutants. The process demonstrates significant removal of suspended materials, colloidal substances of different sizes and even dissolved macromolecules from the liquid portion with excellent efficiency. In the process, a specific solid membrane with a pore size of normally less than 100 nanometers is used to separate particulate contaminants, micro/nanoplastics and viruses from water. However, ultrafiltration is intrinsically associated with two major issues. The first one is the fouling of the membrane due to biotic and abiotic contaminants accumulated on the surface and even in the internal pores of the membrane. Also, the growth of a vibrant cake layer of micro/nanoplastics takes place on the surface of the membrane.^109,110^ Although micro/nanoplastic pollutants are effectively detained and intercepted through the size-sieving ability of the membrane, the accumulated pollutants on the surface of micro/nanoplastics greatly reduce the efficiency of ultrafiltration through fouling, pore filling and pore tapering. The second is the low efficiency removal of hydrophobic contaminants and low-molecular-mass organic compounds.^111^ The process efficiency can be effectively augmented by using certain coagulants before ultrafiltration. To maintain the performance of the process, regular physical and chemical washing of the membrane is required to eliminate the fouling deposits. In physical cleaning, backwashing through reverse flow of water allows the extrication of the gathered foulants and micro/nanoplastics. In chemical cleaning, certain oxidants, acids and bases are used to remove organic pollutants.^112^

Intensive research has been conducted on the preparation of nanostructured membranes, which are further modified to achieve improved surface properties and separation performance. Yang *et al.* synthesized a holey reduced graphene oxide (h-rGO)-based membrane with an average pore size of 27.3 nm for the treatment of microplastics (Fig. 14a and b). The h-rGO membranes removed microplastics in the permeate solution by size exclusion with a removal efficiency of 99.9% for polystyrene beads and fluorescent polystyrene microspheres of varying size. More importantly, when these membranes were tested against real wastewater samples, they continued to work well, capturing almost all the microplastics.^113^ Recently, MOFs have attracted massive attention for the formation of ultrafiltration membranes due to their excellent porosity, customizable pore size, and excellent surface area to overcome the low permeability of water during the filtration process. In the environment, micro/nanoplastics have a positive or negative charge due to the presence of co-existing pollutants of various natures (metals, salts, organic pollutants, *etc.*).^114^ Therefore, ultrafiltration membranes composed of MOFs can be effectively used to remove these plastic materials with high efficiency. The formation of a MOF-based ultrafiltration membrane is shown in Fig. 14c.

**Fig. 14:**
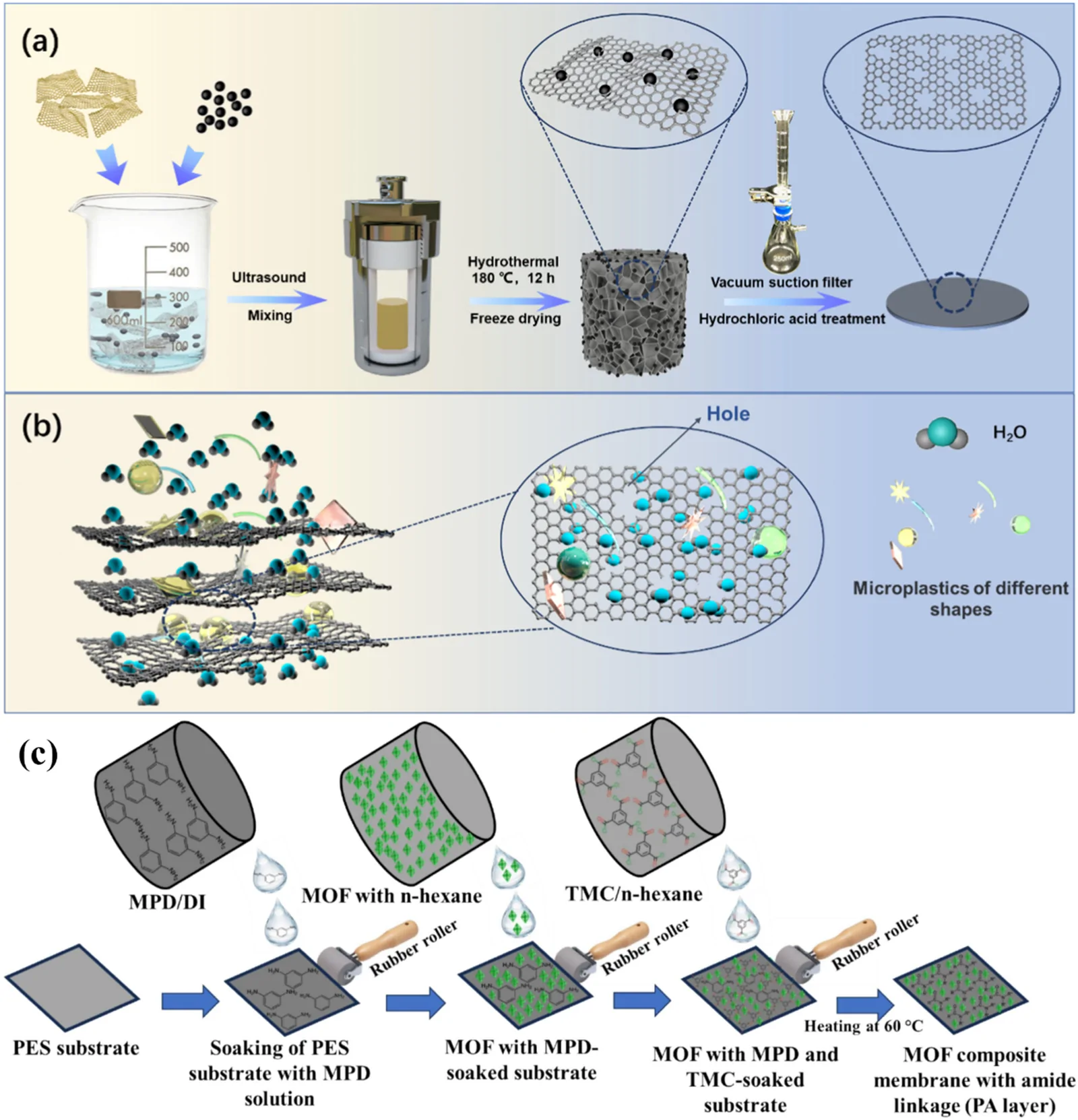
Schematics of (a) the morphological synthesis of an h-rGO based membrane and (b) filtration of microplastics. Figure adapted from ref. 113 with permission from Elsevier, copyright 2025 under License Number: 6340670774522. (c) Synthesis of a MOF-based ultrafiltration membrane (MPD stands for *M*-phenylenediamine, PES stands for polyethersulfone, TMC stands for trimesoyl chloride, PA stands for polyamide, and DI stands for deionized water). Figure adapted from ref. 114 which is open access and allows the use of Figures under the Creative Commons CC BY 4.0.

#### 8.2.4 Froth floatation technique (FFT)

The plastic recycling process is generally composed of four steps: collection, separation, management and sale. Owing to the differences in their physical and chemical properties, mixtures of plastics are not recycled directly, as one plastic generally decreases the quality of the other plastics. The froth floatation technique (FFT) is a valuable physicochemical process used to separate plastic materials for effective recycling. This technique was originally employed to industrially separate minerals of different natures. It is a complex process involving three distinct segments: solid, liquid, and air. FFT starts with size attenuation to separate lighter plastic materials from heavier ones through sink-float separation. The process is realized by passing air bubbles continuously under agitation to form a froth. The basic principle of FFT lies in the selective combination of plastic materials and air bubbles depending on the surface hydrophobicity of the plastic materials. As a result, the hydrophobic particles migrate to surface with air bubbles, while the hydrophilic particles are wet by the liquid, generally water, submerged to the bottom and retained in the liquid portion. Thus, the attached air bubbles transport plastic materials from the bottom to the surface of the liquid, from which they can be skimmed and separated effectively as shown in Fig. 15.^115,116^ A major obstacle in the FFT lies in the selective and efficient wettability of plastic materials. This obstacle may be overcome by lowering the vapor pressure of the liquid to decrease its surface tension. This practice is generally called gamma flotation. Since the majority of plastics are naturally hydrophobic due to small surface energy, their separation through FFT requires initial surface treatment with characteristic reagents to change the surface from hydrophobic to hydrophilic. These are generally traditional reagents such as malic acid, terpenes, dechlorination, metal lignosulfonate, hypochlorite, and ferrate. Several factors, such as the surface chemistry, size, shape and morphology of the plastic materials, control the froth formation. During the aging process, plastic materials lose their intrinsic hydrophobic properties, and therefore, their attachment to air bubbles for effective transportation to the surface for separation becomes easier.^117–120^

**Fig. 15:**
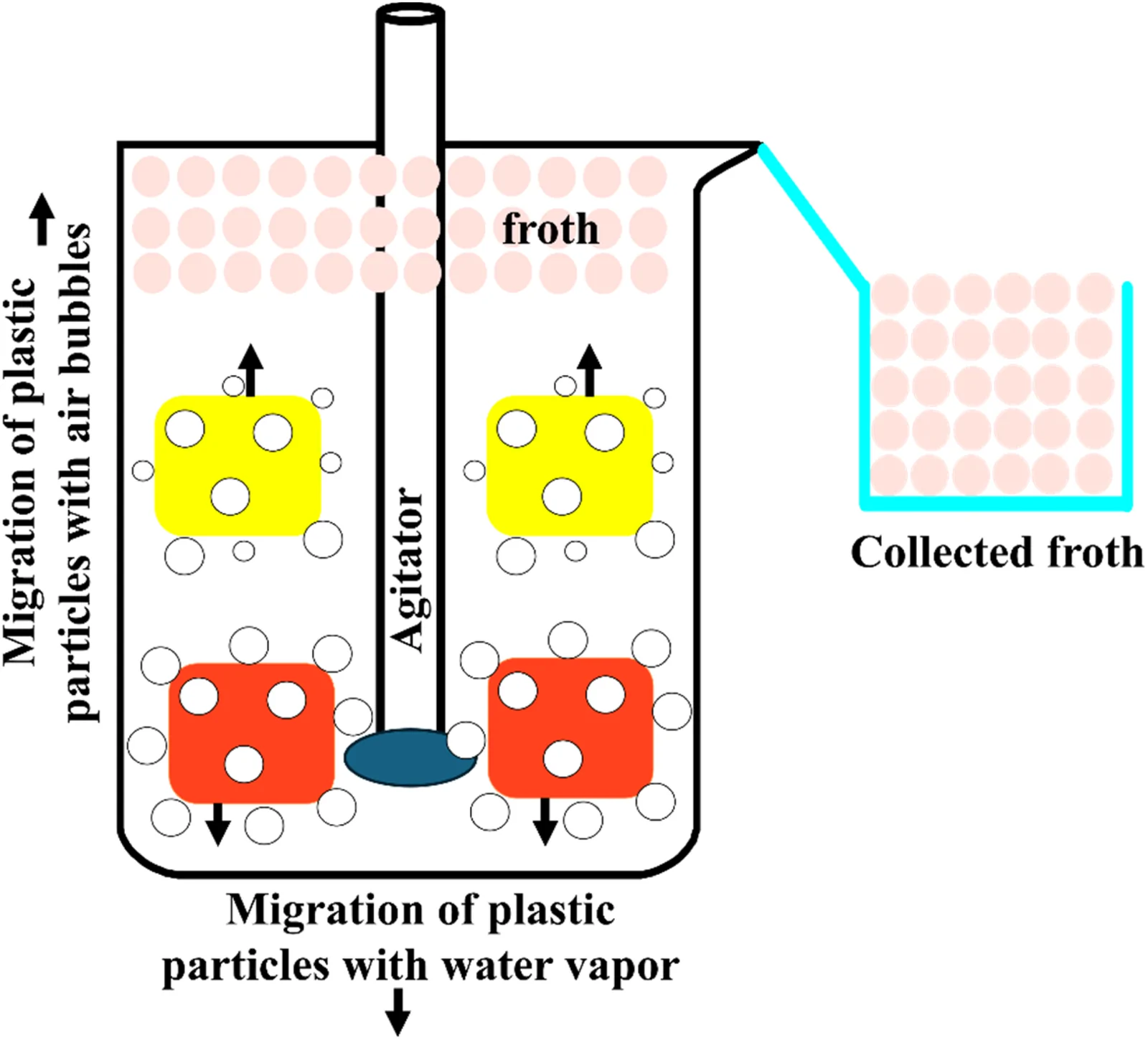
A simple schematic of FFT.

### 8.3 Chemical recycling

As discussed in section 7.1, chemical recycling or advanced recycling is a fundamental technique related to the degradation of plastics into their primary modules. The process is realized by depolymerizing the polymer into its monomers/oligomers through several chemical reactions or into synthetic oil (higher hydrocarbons) through heating in the absence of oxygen in a circular economy. During depolymerization, the target plastics are converted into their monomers/oligomers through chemical reactions, which depend strongly on the type of plastic, type of catalyst and reaction temperature. However, the conversion of plastics into synthetic oil (mainly hydrocarbons) requires heating in the absence of oxygen, for example, the conversion of plastics to energy through combustion/gasification, the conversion of plastics to CH_4_ and hydrogen through gasification, and the conversion of plastics to oil by thermal pyrolysis in a circular economy. This technique significantly relieves the environmental liabilities connected with plastic pollution and has climate benefits. A basic assumption of the circular economy is the protection of the structural integrity of the recycled plastic as supported by chemical recycling.^121^ Several chemical procedures have been adopted to reduce the impact of plastic pollution, such as ozonation, coagulation, electrocatalysis and photocatalysis, as shown in Fig. 16. In the following sections, we have discussed these techniques in detail.

**Fig. 16:**
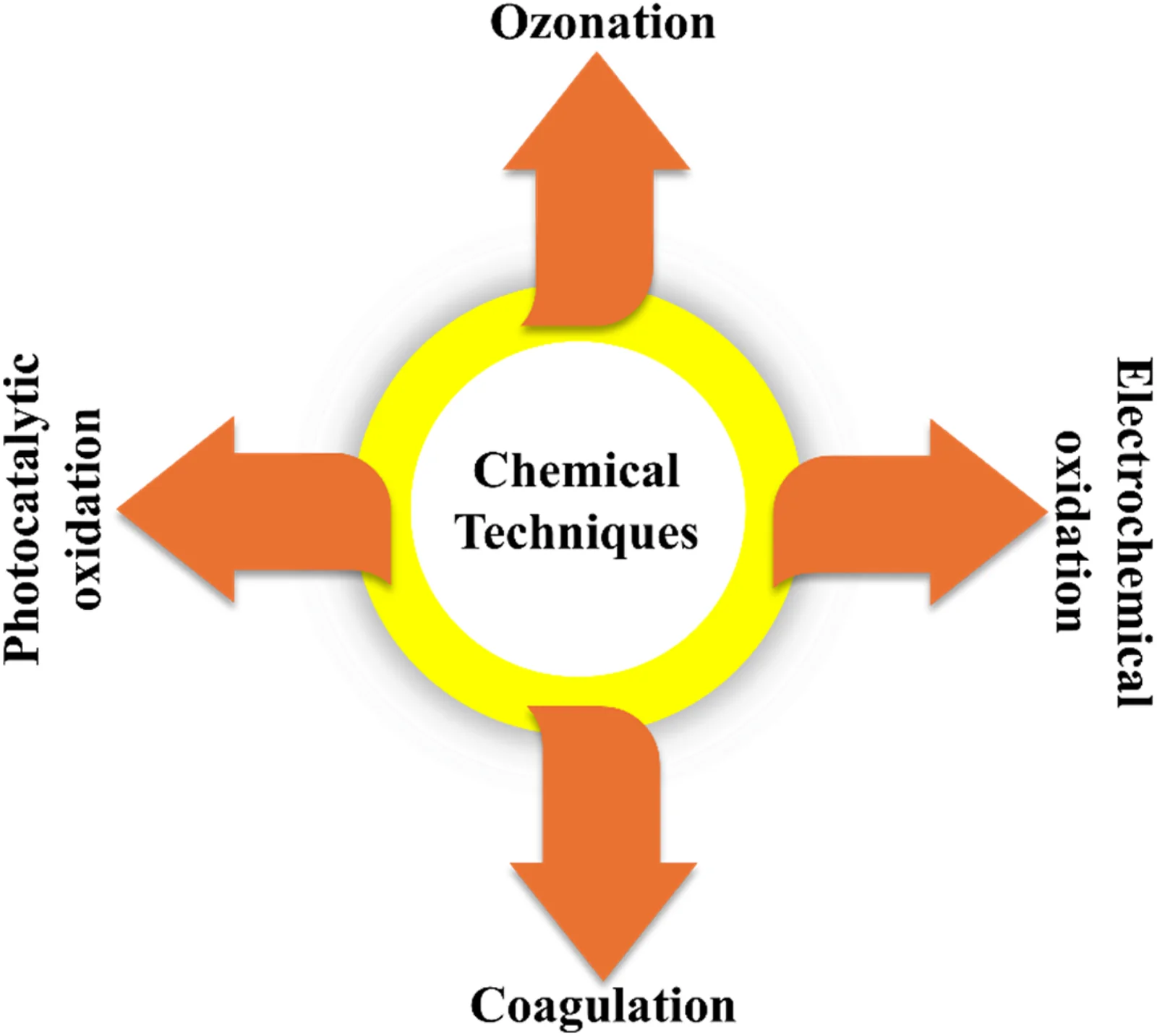
Chemical techniques for the eradication of micro/nanoplastics.

#### 8.3.1 Ozonation

Ozone (O_3_) is an allotropic form of oxygen. It is a highly reactive allotrope and is extensively employed to decompose organic pollutants in water. The ozonation process is substantially oxidizing under a controlled operational environment.^122^ It attacks the chemical structure of micro/nanoplastics, resulting in their degradation and a significant reduction in their lifetimes. The removal efficiency is proportional to the pressure of the gas, although low quantities of ozone can produce exceptional degradation at room temperature over time.^123^ The process results in the formation of new chemical bonds by altering the bond dissociation energies and adding new oxygen functionalities (aldehydes, ketones, alcohols, and carboxylic acid). The introduced functionalities improve the hydrophilicity of the plastic to promote further degradation. Ozonation is employed as a pretreatment technique to alter the chemical structure of micro/nanoplastics to make them more approachable for microbial degradation. During ozonation, certain toxic compounds are also generated, especially when the micro/nanoplastics are loaded with organic wastes, generating a possible threat to the environment.^124^

Although ozonation is exceptionally effective in the decolorization and sterilization of drinking water, the process is not very efficient for the elimination of plastic materials.^125^ The process is realized *via* homogeneous catalytic ozonation, in which transition metal ions act as catalytic centres and heterogeneous catalytic ozonation, in which solid transition metal oxides act as catalytic centres. The disadvantage of homogeneous catalytic ozonation is that the recovery of metal ions is difficult, causing secondary pollution, while heterogeneous catalytic ozonation is relatively safe and more effective.^126,127^

In the ozonation of polystyrene, several organic compounds, such as HCOOH, C_6_H_5_OH, hydroquinone, acetophenone, methyl acetophenone, methyl benzaldehyde, phenyl propionic acid, aldehydes and ketones, were detected, demonstrating the conversion and degradation of polystyrene, as shown in Fig. 17. The attack of ozone initially resulted in the formation of hydroperoxide followed by its conversion into acetophenone, benzaldehyde, styrene oxide, *etc.*^128^ The degradation of micro/nanoplastics results in the release of various chemical compounds such as antioxidants, plasticizers, flame retardants (brominated compounds), and stabilizers to the environment. Interestingly, these compounds, which are added to the polymers to improve their flexibility and stability, are not chemically attached to the plastic materials. Generally, bisphenol A, phthalates, and nonylphenols are released in detectable quantities into the external environment, causing secondary pollution.^129^

**Fig. 17:**
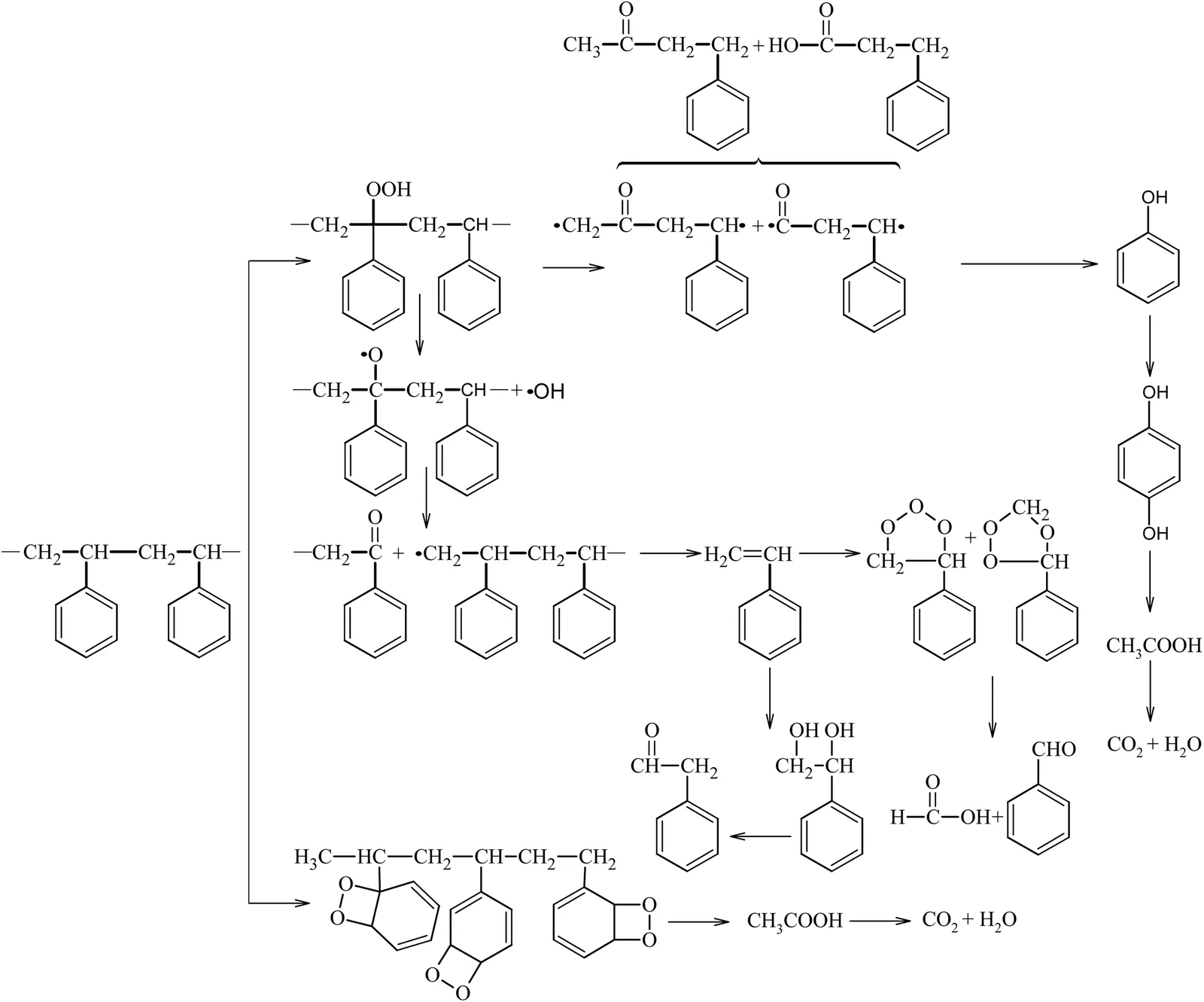
Degradation pathways of nano-sized polystyrene plastics through ozonation. Figure adapted from ref. 128 with permission from Elsevier, copyright 2025 under License Number: 6340671294933.

#### 8.3.2 Coagulation

The removal of micro/nanoplastics by chemical methods has become a major priority recently, as these methods have shown significant results in the removal of hazardous materials on an industrial scale. Although chemical methods deliver significant efficiency, their high energy requirements, environmental sustainability, and formation of secondary pollutants still remain to be tackled to confirm their wide-range applicability in wastewater treatment. Coagulation is a simple chemical method in which certain chemicals, called coagulating compounds or coagulants, are added to water containing micro/nanoplastics to affect their polarity *via* a charge neutralization mechanism. As a result, the agglomeration and aggregation of micro/nanoplastic particles take place to form a mass called coagulum or floc. The sedimentation of coagulum significantly promotes the separation of pollutants from testing liquid medium.^130,131^ The use of coagulation as a pollutant eradication technique has attracted massive research due to its low working cost, reduced carbon footprint, and environmentally friendly features. Several chemicals have been used as coagulants to change the physicochemical characteristics of plastic materials for facilitation of their removal. The efficiency of coagulation is a function of the coagulant type, adsorption, and sweep flocculation, as shown in Fig. 18.^132^ Generally, salts of Al and Fe are used to accelerate the coagulation and removal of plastics from water. AlCl_3_, FeCl_3_, and Al_2_(SO_4_)_3_⋯18H_2_O, frequently called coagulant alum, are used to coagulate plastic materials into flocs for their removal. However, Al salts are more effective than Fe-based coagulants particularly in the removal of weathered plastics, as weathering significantly changes the physical/chemical properties of plastics to improve their interaction with coagulants.^133,134^ In order to improve the performance of coagulants, certain additives called flocculants or coagulant aids are introduced along with the usual coagulants.^135^

**Fig. 18:**
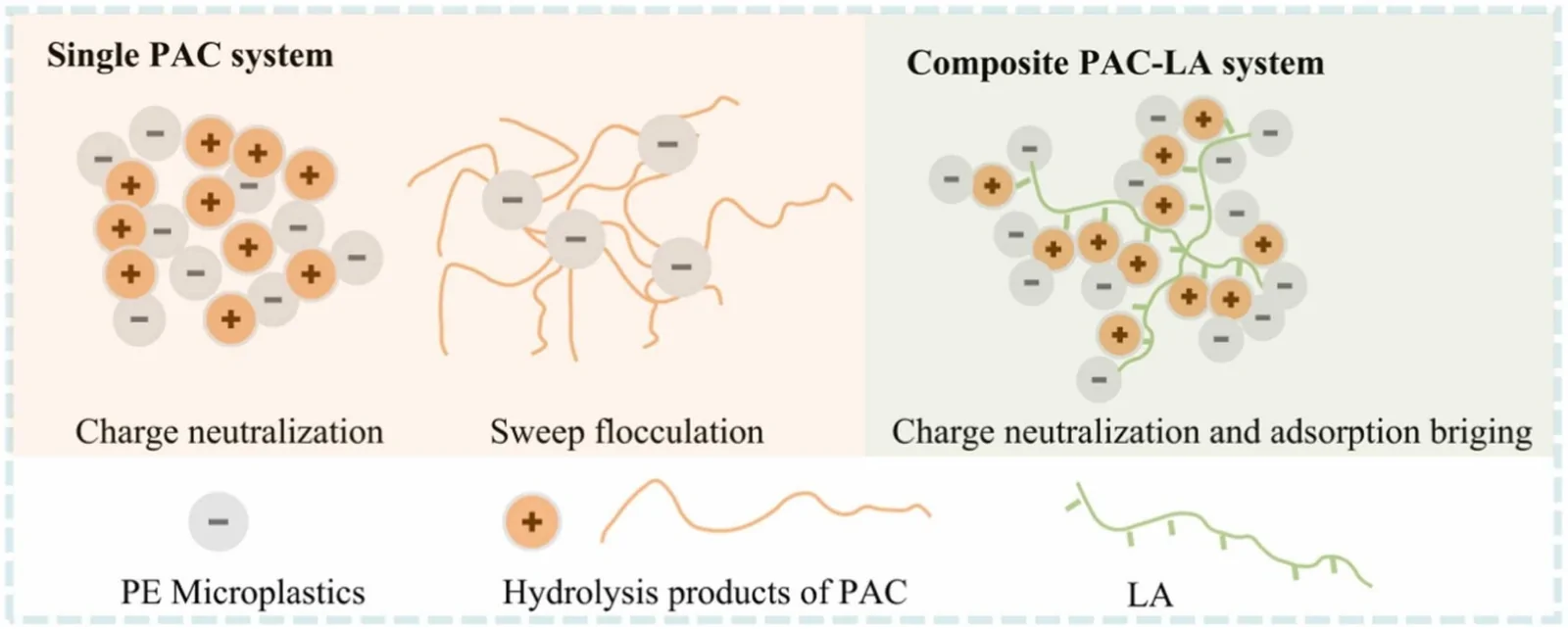
Coagulation mechanism for the removal of plastics. Figure adapted from ref. 132 with permission from Elsevier, copyright 2025 under License Number: 6340680121691.

In coagulation, several mechanisms, such as charge neutralization, adsorption and sweep flocculation, are involved in the removal of micro/nanoplastics. The surface of plastics is negatively charged due to oxygen functionalities. As a result of the electrostatic repulsion, micro/nanoplastics are well distributed in water, thereby making their removal more difficult. During the charge neutralization process, the negatively charged surface is neutralized and counterbalanced by the positively charged metal ions or hydrolysis products (hydrolysates) of the coagulants. The positively charged metal ions and hydrolysates adsorb on the superficial surface of the micro/nanoplastics to form agglomerates *via* electrostatic repulsion elimination. As a result, the well-dispersed particles come close to each other and agglomerate to form floc for removal.^136,137^ Sweep flocculation takes place when the density and size of flocs increase with time due to adsorption of the micro/nanoplastics, leading to sedimentation and precipitation. Adsorption of coagulants on the surface of the micro/nanoplastics takes place through hydrophobic and electrostatic attractive forces assisted by hydrogen bonding.^138,139^

Although the optimum amount of coagulant is different for different plastics, it is accepted that increasing the amount of coagulant generally increases the removal efficiency.^140^ It is found that inorganic coagulants are less effective than organic and biological ones, as the hydrolysis of inorganic metal cations requires large hydration energy. The interaction of metal ions with the micro/nanoplastics becomes difficult due to their encapsulation by water molecules through metal–ligand (polar water) interaction. However, organic and biological coagulants have large molar masses with exceptional charge densities. The large polymeric complex structures of these coagulants remove micro/nanoplastics exceptionally well through the adsorption bridging technique. On the same grounds, coagulants with high charge density strongly facilitate the removal of micro/nanoplastics through their excellent capability to impart more charge density to the plastic materials during the neutralization process.^141^ However, coagulation also has many shortcomings. The introduction of chemicals in the form of coagulants results in the generation of some undesirable hazardous byproducts, which become difficult to remove.

#### 8.3.3 Electrocatalytic oxidation

Plastic pollution is generally treated through landfilling and incineration. However, these techniques pose grave health and environmental issues. Landfilling is a waste of valuable land and resources, and introduces micro/nanoplastics to the soil through leaching, while incineration results in the accumulation of dioxin-like carcinogenic and toxic gases in the atmosphere. It should be remembered that upcycled plastic is substantially inferior in quality compared to the original products.^142,143^ The electrochemical conversion of plastic into value-added compounds represents a unique cutting-edge technique that realizes electrochemical reactions through the application of an applied current. It is a promising and sustainable technique, and enables electrodes (catalysts) to expedite redox reactions under a milder and controlled environment, generally in combination with renewable energy for the selective degradation of plastics. Electrochemical upcycling involves key steps such as electron transfer processes, surface catalysis and depolymerization of the polymeric structure to form useful compounds.^144^

Encouraged by the electrochemical conversion of organic compounds into value-added products, researchers have found that organic polymers can be selectively depolymerized and upcycled through activation of carbon–carbon bonds by inducing electrical energy in the presence of suitable working electrodes. Generally, electrochemical water splitting involves two half-reactions, the anodic half-reaction evolving oxygen gas and the cathodic half-reaction evolving hydrogen gas. However, the anodic half-reaction is sluggish, and requires high energy input to proceed with comparable rates. By coupling with the conversion of waste plastics, the electrochemical conversion is made exceptionally valuable, producing hydrogen cathodically and eradicating pollutants anodically.^145^ The electrochemical conversion of plastics is generally carried out in three major steps. In the first step, the polymeric structure is broken down into its precursor monomers (conversion of polyethylene terephthalate to terephthalic acid and ethylene glycol) through hydrolysis. In the second step, oxidation of the formed intermediates takes place to form value-added products such as formate and glycolic and succinic acids. In the third step, formate and glycolic acid are further converted selectively into more beneficial products. As shown in Fig. 19a, when current is passed through an electrolyte solution containing plastics, the adsorbed oxygen gas and water molecules are converted to form degrading species.^146^

**Fig. 19:**
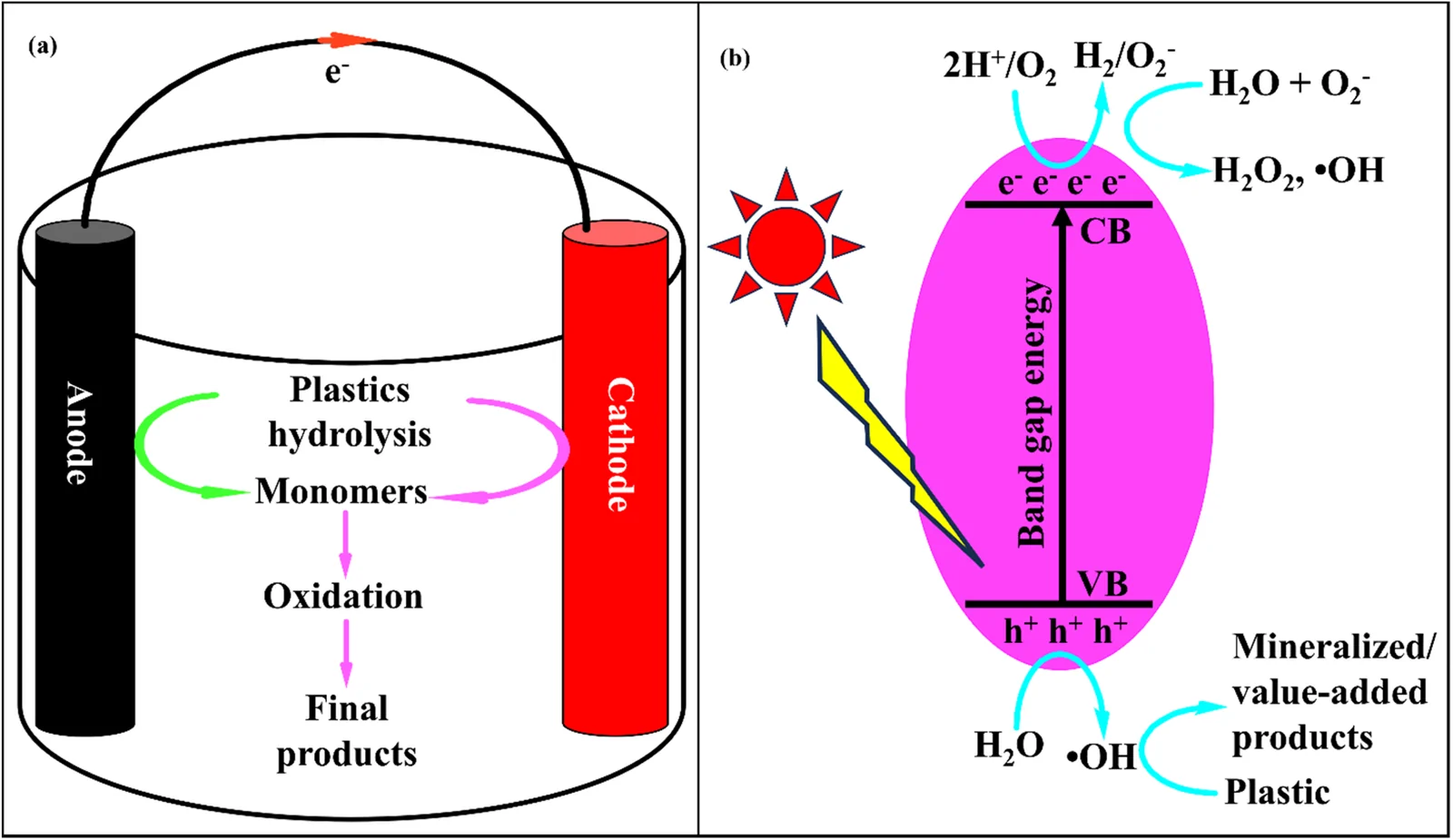
(a) Electrochemical degradation of micro/nanoplastics and (b) generation of excited electrons and holes for the production of reactive oxygen species and degradation of plastics.

Electrochemical upcycling of plastics offers several distinctive benefits over physical and biological processes. The key advantage is the low energy requirement. The process generally uses the chemically stored energy in the hydrolytic products (ethylene glycol) to form formate and hydrogen gas under low energy requirements. The process presents substantial selectivity in the degradation of plastics. The traditional techniques result in the formation of several products, which are difficult to separate, requiring further treatment, labour and energy. The electrochemical technique can be modulated to generate selective and value-added energy products such as formate and hydrogen gas with excellent faradaic efficiency.^147^

The low chemical reactivity and poor solubility of plastics in water present serious challenges in their direct electrochemical conversion. Several key parameters, including the selection of electrodes, nature of the electrolyte, magnitude of the applied current and reaction conditions play significant roles in the overall efficiency of the process.^148^ The nature of the electrolytic solution and electrodes has vast effects on the faradaic efficiency of the electrochemical process. For electrodes, the electronic structures must be precisely modulated to control the adsorption of micro/nanoplastics followed by their activation and conversion into energy-rich substances. Porous electrodes with more active sites are very impressive for the adsorption of micro/nanoplastics and desorption of key intermediates. Aqueous solutions of electrolytes are widely employed to meet economic demands. Some organic compounds and ionic liquids are also used to improve the solubility of plastics for fast reaction kinetics. The selectivity of the electrodes also dictates the plastic removal efficiency. Generally, B-doped diamond and iridium oxide-based anodes provide excellent performance to generate reactive oxygen species for the degradation process^144,149^^.^

#### 8.3.4 Photocatalytic oxidation

Plastic-based pollution is of major concern for the following reasons: (a) plastic possesses a long-chain polymeric structure formed by the addition or condensation reactions of thousands of monomers. This long polymeric structure makes plastic highly persistent in the environment. (b) Various additives, such as stabilizers, heavy metals, plasticizers, and foaming agents, are added during the manufacturing of plastic, which further intensifies environmental pollution. (c) The wasted plastic undergoes transformation due to various factors (wind, pressure, moisture, oxygen, microbial activity, solar light, mechanical stress, *etc.*) to form micro/nanoplastics, which endanger terrestrial and aquatic life. Therefore, advanced techniques to deal with plastic pollution are of prime importance.^150^

The photocatalytic removal of pollutants has been developed as a favorable solution due to its low cost and high efficiency. This technique utilizes green energy to reduce reliance on traditional carbon-based fuels. The photocatalytic removal of pollutants involves several steps.^151^ The photocatalytic process commences when solar light of suitable energy is absorbed by the working material, called the semiconductor or photocatalyst. The absorbed light results in the generation of photoexcited electrons and holes, which separate and migrate to the surface of the photocatalyst for redox reactions.^152,153^ These excited charges either directly attack the target pollutants or undergo chemical reaction with absorbed oxygen and water to form superoxide anions (˙O_2_^−^), singlet oxygen (^1^O_2_) and hydroxyl free radicals (˙OH). These generated degrading species work to break the long polymeric chains due to their oxidizing nature. The photogenerated free radicals have two modes of action on the surface of plastic. In direct attack, the oxygenated degrading species interact with the surface of plastic to form various intermediates, followed by their conversion into value-added products or water and CO_2_. In indirect attack, since plastic is a home for the accumulation of various compounds and elements present in water, the photocatalytically formed oxygenated species react with these compounds to form new free radicals on the surface of or inside the plastic. The newly formed radicals and intermediates then attack the weak bonds in the polymeric structure to complete the degradation process. The specific locations at which free radicals attack depend on the nature of plastic. Normally, the radicals trigger chain-scissoring reactions when they come in contact with non-polar plastics, while they tend to attack the vicinity of functional groups in polar plastics.^154,155^ As a result, plastic is ultimately converted into low-molar-mass compounds or completely mineralized into H_2_O and CO_2_, as shown in Fig. 19b.

Broadly, the photocatalytic conversion of plastic can be classified into complete photo-oxidation, selective photo-oxidation and photoreforming. In complete oxidation, the plastic-based materials are oxidized into water and carbon dioxide by the excited charges. Both electrons and holes take part in the degradation and mineralization of plastic into H_2_O and CO_2_. In selective oxidation, the plastic-based materials are converted into several oxygenated compounds owing to the application of reactive degrading radicals such as superoxide anions, singlet oxygen and hydroxyl free radicals.^156^ In photoreforming, the excited holes in the valence band of the semiconductor are used to oxidize pretreated micro/nanoplastics, while the electrons are left for the reduction of water to produce hydrogen gas. Complete mineralization and selective oxidation are classified as indirect and direct upcycling techniques, respectively. The intermediates formed during the selective oxidation of plastic serve as precursor building blocks for the production of value-added compounds. Due to its dependence on multiple sequential steps of photocatalysis, this route is considered an indirect process. The photocatalytic conversion of plastic into harmless compounds is further accelerated under oxygen-rich conditions (called oxygen production reactions), as more superoxide anions are formed, which upon reaction with water produce ˙OH radicals. Thus, this technique not only removes micro/nanoplastics, but also obtains beneficial compounds, providing a viable substitute to traditional plastic removal techniques.^157,158^

The rate of photocatalysis also depends on the nature of the semiconductor and charge separation. Semiconductors with more active sites and low bandwidths are superior for the effective degradation of plastic. Highly separated excited electrons and holes have more time to attack the polymeric structure for redox reactions, while semiconductors with high recombination of electrons and holes realize sluggish photocatalytic kinetics during the removal of plastics. The wavelengths of the incident light and its intensity have pronounced effects on the efficiency of photocatalysis. Light with shorter wavelengths is more energetic, producing large numbers of excited electrons and holes for the degradation process, as can be seen in case of UV-stimulated semiconductors. Similarly, light with high intensity increases the number of excited electrons and holes for the degradation process.^159^ The chemical structure of the plastics also influences their photocatalytic removal efficiency. Micro/nanoplastics with a hydrophobic surface only partially interact with water and generated oxygen species, and therefore, their removal rate is slow compared to micro/nanoplastics having hydrophilic surfaces that adsorb water molecules and hydroxyl free radicals more effectively for their accelerated removal.^160^ For instance, Sarwan *et al.* used BiOCl nanoparticles for the degradation of polystyrene.^161^ The excited electrons generated superoxide anions upon reaction with adsorbed oxygen molecules, while the holes in the valence band formed ˙OH radicals with water. These species degraded plastic into several intermediates, followed by their conversion into aromatic aldehydes and carboxylic acids. The aromatic compounds were mineralized into water and CO_2_ upon reaction with the generated species, as shown in Fig. 20.

**Fig. 20:**
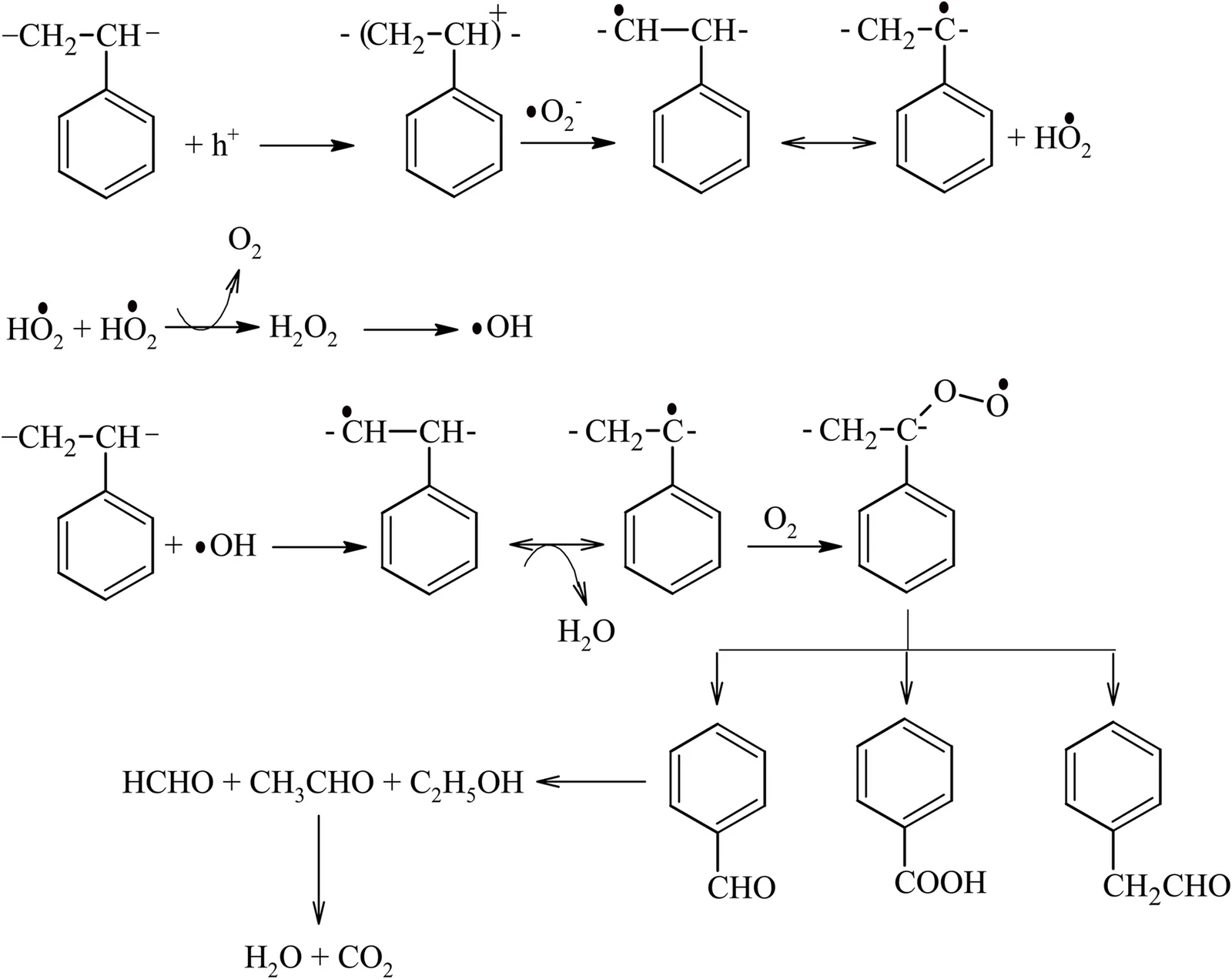
Mineralization of polystyrene plastic under solar-light-driven photocatalysis.

Currently, various catalysts such as zeolites, and metal oxides are commonly used to deal with plastic-based pollution owing to their high removal efficiency and stability. However, these catalysts also face many hurdles. For the photocatalytic removal of micro/nanoplastics, the mesoporous structure of biochar is very conductive to the interaction of plastic particles with the active sites of biochar, mass transfer and diffusion of the formed energy-rich compounds. Biochar-based photocatalysts are exceptionally stable, chemically inert and easily biodegradable. Biochar significantly improves optical absorption and boosts the performance of the semiconductor. During the photocatalytic conversion of plastics, the separation of excited electrons and holes is crucial, as these species intrinsically tend to undergo recombination and waste time. The separation of these charges can be boosted by introducing solid biochar-based materials in the formulation of the photocatalysts. The large surface area of biochar significantly improves the distribution of particles and prevents their aggregation. The excellent dispersion increases the number of active sites, enhancing the scattering of irradiated light for improved removal of plastics. Additionally, the presence of certain functional groups of biochar helps to improve the adsorption of plastics and their conversion to value-added products. A photocatalyst with a porous structure and large number of active sites has many advantages, such as improved light absorption due to manifold scattering due to porosity, good dispersion of particles to reduce charge diffusion length, promotion of excited charge separation, generation of a built-in electric field in the radial direction of the pores, and high density of active sites due to high surface area. To further improve the pollutant removal efficiency of biochar-based photocatalysts, many authors have treated biochar with strong acids, doped it with active metals and coupled it with co-catalysts.^162,163^Table 1 presents the summary of the different techniques and their efficiencies in the removal of plastic.

**Table 1:** Different techniques used for the removal of plastics and plastic additives

Technique	Materials/bacteria used	Materials removed	Efficiency	Ref.
Adsorption	Activated charcoal	Mixture of dye and polystyrene	99%	164
Adsorption	Zn/Al layered double hydroxides	Nano-scale plastic debris	100% at pH 4	165
Density separation	Water	Polystyrene, polymethyl methacrylate, polyethylene terephthalate and polyvinyl chloride	85.1% for different polyvinyl chlorides	166
Adsorption	Surface-functionalized cellulose fibers	Polymer nanoparticles	>98%	167
Biodegradation	*Pseudoxanthomonas* sp. strain NyZ600	Bisphenol-A polycarbonate plastic	—	168
Adsorption	Magnetic-activated biochar	Bis(2-ethylhexyl) phthalate and di-*n*-butyl phthalate plasticizers	89.31% and 41.25%, respectively	169
Ozonation	CeO_*x*_@MnO_*x*_ catalyst	Polystyrene	96.70% molecular weight degradation in 50 min	170
Ozonation	Fe^3+^, Co^2+^, Ni^2+^, and Zn^2+^	Polystyrene	A rapid size reduction of >99% in less than 5 min	171
Adsorption	Multi-wall carbon nanotubes and magnetic Fe_3_O_4_	Dimethyl phthalate plasticizer	196.85 and 136.99 mg g^−1^, respectively	172
Pyrolysis and adsorption	Red mud or bauxite residue	Polyethylene	—	173
Biodegradation and adsorption	Marine suspended particulate matter and various types of nano-TiO_2_	Bisphenol A, 4-*tert*-octylphenol, 4-nonylphenol	Biodegradation efficiency of 25, 9 and 2%, respectively, and 37, 75 and 100% adsorption capacity, respectively	174
Biodegradation	Microorganisms	Polybutylene sebacate	100% in days	175
Co-pyrolysis and catalytic processes	Zeolite ZSM-5 catalyst	Polyethylene, polypropylene, polystyrene and polyethylene terephthalate	—	176
Froth floatation	Tannic acid as a wetting agent	Polystyrene, polymethyl methacrylate, polyethylene terephthalate and polyvinyl chloride	95.9% for polystyrene	177
Density separation	CaCl_2_-water solution	Polyethylene terephthalate and polyvinyl chloride	83%	178
Density separation	Hydrocyclone, DMS cyclones, LARCODEMS cyclone	Flaky plastic particles of varying size	57.7 and 100%, respectively, for hydrocyclone and LARCODEMS cyclone	179
Advanced oxidation process	Amorphous B as an activator of peroxymonosulfate	Bisphenol and phthalate plasticizers	95.0, 67.4, 18.3 and 0.6%, respectively, in ultrapure, tap, sea and river water for diethyl phthalate	180
Density separation	Water	Polypropylene and high-density polyethylene	100%	181
Froth floatation	Potassium permanganate	Polycarbonate, polyvinyl chloride and polymethyl methacrylate	96.82, 98.71 and 98.38% for polycarbonate, polyvinyl chloride and polymethyl methacrylate, respectively	116
Ozonation	MnO_2_, oxalic acid and O_3_ system	Polystyrene	TOC removal of 75%	182
Ozonation	O_3_/H_2_O_2_	Polyethylene, polypropylene, polyvinyl chloride, and polystyrene	Loss of surface area was in the range of 1.3 to 26.7%	129
Froth floatation	Potassium hydroxide and ethylene glycol	Polyethylene terephthalate, polycarbonate, acrylonitrile–butadienestyrene copolymer, and polyvinyl chloride	98.8% for polyethylene terephthalate recovery	183
Membrane separation	Composite membrane of polyacrylonitrile and reduced graphene oxide	Microplastics	Rejection > 82%	184
Membrane, air flotation and nano-ferrofluid processes	—	Polyethylene, polyvinyl chloride and polyester	85, 82 and 69% for polyethylene, polyvinyl chloride and polyester, respectively	185
Ozonation	O_3_/ultrasonic/UV oxidation	Low-density polyethylene	The efficiency was 3.84 times greater than O_3_ alone	186
Ozonation	O_3_	Nano-sized polystyrene	99.9% molecular weight degradation and 42.7% mineralization in 240 min	128
Coagulation	Fe(ii) and Al(iii) sulfates	Polystyrene and polyvinyl chloride	99 and 100% for polystyrene and polyvinyl chloride, respectively	187
Coagulation	Tannic acid modified FeCl_3_	Polystyrene	90% in 5 min	188
Ozonation	O_3_	Polyethylene	92%	123
Ozonation	O_3_	Low-density polyethylene, polypropylene, and polyamide 66	—	189
Ozonation and flocculation	O_3_, aluminum sulfate and polyacrylamide	Polyvinyl chloride and polyethylene terephthalate	After ozonation, the removal efficiency enhanced by flocculation from 40 to 91%	190
Coagulation	Chitosan as a coagulant	Polyethylene	81.5% at a pH of 6	191
Electrocoagulation	—	Polyvinyl chloride, polystyrene, polypropylene, and polyethylene	77.2, 74.0, 70.8, and 69.2%, respectively	192
Coagulation	Polyaluminum chloride, ferric chloride, aluminum chloride, alum and aluminum sulfate	Polyamide, polystyrene, and polyethylene	65, 22, and 12% for polyamide, polystyrene, and polyethylene, respectively	193
Coagulation	Magnetic magnesium hydroxide and non-ionic polyacrylamide as a coagulant	Polyethylene	92%	194
Coagulation	Aluminum chlorohydrate and polyacrylamide	Polystyrene	98.5% at a pH of 8	195
Coagulation	Ferric and aluminium sulphate	Polyvinyl chloride	80%	196
Photocatalytic oxidation	Ni clusters decorated TiO_2_ nanosheets with oxygen vacancies	Polyethylene	—	197
Photocatalytic oxidation	WO_3_ nanoflower	Polyethylene	—	198
Photocatalytic oxidation	ZnS/Ga_2_O_3_*Z*-scheme heterojunction photocatalyst	Polyethylene	—	199

## 9 Conclusion

Pollutants of diverse chemical nature have caused major issues in both plants and animals. The deterioration of the environment due to the presence of plastic is a hot issue currently. Organisms of diverse habitats are affected due to the presence of micro/nanoplastics in water, and soil. Here, we have discussed the applications of plastics, particularly micro/nanoplastics, in detail. The effects of micro/nanoplastics on soil, crop productivity, aquatic animals, and human beings have been elaborated in detail. Physical and chemical recycling processes and various eradication techniques to cope with plastic-based pollution and its impacts on society, including adsorption, density separation, ultrafiltration, froth floatation, ozonation, coagulation, and electrocatalytic and photocatalytic oxidation, have been discussed. We have also provided a bird's-eye view of the role of biochar in the adsorptive and photocatalytic removal of micro/nanoplastics. We hope that this article will provide significant research directions for dealing with plastic-based pollution.

## 10 Future directions and perspectives

Currently, plastic is a crucial part of our life. The uses of plastics in shelter, food, clothing, medicine packaging, medical instruments, vehicles, and aeroplanes are countless. Because of these exceptional applications, an extraordinary wave of plastic production in the last few decades has caused widespread environmental pollution. It has migrated everywhere on the globe and is frequently found in water, soil, and even in the bodies of animals, plants and microorganisms. Plastic pollution manifests as large visible particles as well as micro/nanoplastics, which can penetrate the cells and tissues of animals and plants, resulting in damage and injury, inflammation, swelling, infection and even cardiovascular issues. Owing to its hazardous effects, dealing with plastic pollution is essential to prevent its excessive accumulation in soil and water.

The accumulation of micro/nanoplastics in different segments of the environment has been studied by several authors. However, their origin, distribution, fate and removal are still mostly undiscovered. Although various traditional techniques, as well as advanced oxidation methods, have been employed to investigate the removal of micro/nanoplastics, these techniques are not efficient enough to remove and deal with the already diversely distributed plastics. Additionally, these techniques are costly and ecologically not maintainable to reach the goal of a sustainable environment. Due to the great challenges and limitations in the management of micro/nanoplastics, it is very difficult to achieve the complete removal of plastics.

Extensive research is required to find truly biodegradable polymers that completely break down in soil after a specific lifetime. The use of easily biodegradable polymers must be strictly implemented to provide a good possibility for moving towards reduced environmental hazard. Further, well-organized disposal techniques such as anaerobic breakdown and digestion will significantly reduce the uncontrollable accumulation of micro/nanoplastics.

Future study should prioritize the application of standardized analytical techniques for the quantification of micro/nanoplastics in diverse samples. For this, policymakers should establish concentration limits for micro/nanoplastics in water, and soil samples based on risk assessment thresholds. Standard film thickness and film recovery rates should be validated. Mechanical recovery equipment should be used during harvesting periods. Soil organic matter should be maintained to immobilize plastic particles and trap them in surface soil through incorporation in biochar-based materials. Advanced oxidation processes are expected to play a highly important role in the conversion of plastics into value-added chemicals such as hydrogen, hydrocarbon fuels, and high-cost organic compounds. However, the efficiency of these processes is still low due to the lack of efficient catalysts. Integration of AI-based process optimization and reactor design should be developed to improve the scalability and industrial application of these techniques. The combination of several recycling techniques and use of the principles of circular economy will stimulate efficient removal of plastics with minimum environmental impacts. The impact of various environmental factors, such as different salt ions, temperature, pH, and organic compounds, on the efficiency of different eradiation techniques should be examined in natural soil and aquatic environments, where conditions are highly complex.

Mass awareness is very important to restrict the use of plastics and replace them with easily biodegradable bags for daily uses. Educating the young generation and teaching environmental courses in school and colleges will help to reduce the addition of more plastic-based materials to the environment. Education campaigns will encourage the individual and community to adopt action-oriented techniques for the reduction of plastic use. Awareness of the hazardous effects of plastics must be spread through civic and religious participation, and electronic and print media. This will also help with a cultural shift towards reusing plastic-based items. Although complete removal of the existing micro/nanoplastics in the environment is not possible, a zero-micro/nanoplastics future demands responsible production and management of plastics worldwide and proper dumping and recycling of waste plastics through circular and reformative techniques. This will be achieved through coordinated roadmaps and global collaboration, policy restructuring, new infrastructure growth, and behavioral change to move from plastic-based items to biodegradable ones. In parallel, artificial intelligence must be introduced to track the path and fate of plastics for enhancement of transparency and accountability in the supply chain of plastic-based items.

As far as the biological, physical and chemical removal of plastic is concerned, various initiatives must be adopted to accelerate the degradation process. Emerging techniques based on bioinspired catalysis show appealing trends, offering a responsible solution for the removal of micro/nanoplastics. Based on the inherent stability of polymers, their biological removal still requires controlled pre-thermal and chemical treatments, advanced sorting, refinement, and size alternation before subjecting them to biodegradation. Excessive thermal and chemical treatment before the initiation of biodegradation will weaken the polymeric bonds and ensure fast reaction (enzymatic) kinetics. Enzyme engineering must be coupled with AI to find a broader pH range for the working microbes. The coupling of biodegradation techniques with physical and chemical methods will ensure the release of valuable basic monomers from the wasted polymers, which will strongly support a closed-loop synthesis economy.

The efficient photocatalytic removal of plastics requires significant breakthroughs in the design of photocatalysts and process integration. The hydrophobic nature of plastics hinders their interaction with the degrading species produced during photocatalysis. To realize the selective degradation of polymers, it is critical to understand the process mechanism, especially the generation of intermediate species. Biochar efficiently improves solar light absorption through multiple scattering, accelerates charge separation and increases the number of active sites for the adsorption of reacting substances. To further improve the bioactivity and photocatalytic utility of biochar, virgin biochar can be modified with certain chemicals and additives to improve surface functionalization and pore structures. Like biodegradation, prior thermal and chemical treatment of plastics certainly accelerates their photocatalytic conversion into value-added products.

Future studies must combine environmental assessment with cost and market analyses. These studies may include the composition of plastic, contamination, energy demand, and by-product generation. Studies of recycled plastic must report durability and safety requirements. Therefore, future work must present the sensitivity of the feasibility of recycling to the cost of feedstock in a circular economy.

## Conflicts of interest

The authors declare no conflict of interest.

## Data Availability

No new data were generated in this review.
